# Multi-Ethnic Analysis of Lipid-Associated Loci: The NHLBI CARe Project

**DOI:** 10.1371/journal.pone.0036473

**Published:** 2012-05-21

**Authors:** Kiran Musunuru, Simon P. R. Romaine, Guillaume Lettre, James G. Wilson, Kelly A. Volcik, Michael Y. Tsai, Herman A. Taylor, Pamela J. Schreiner, Jerome I. Rotter, Stephen S. Rich, Susan Redline, Bruce M. Psaty, George J. Papanicolaou, Jose M. Ordovas, Kiang Liu, Ronald M. Krauss, Nicole L. Glazer, Stacey B. Gabriel, Myriam Fornage, L. Adrienne Cupples, Sarah G. Buxbaum, Eric Boerwinkle, Christie M. Ballantyne, Sekar Kathiresan, Daniel J. Rader

**Affiliations:** 1 Broad Institute, Cambridge, Massachusetts, United States of America; 2 Massachusetts General Hospital, Boston, Massachusetts, United States of America; 3 Harvard Medical School, Boston, Massachusetts, United States of America; 4 University of Leeds, Leeds, United Kingdom; 5 Montreal Heart Institute, Université de Montréal, Montreal, Canada; 6 University of Mississippi Medical Center, Jackson, Mississippi, United States of America; 7 The University of Texas Health Science Center at Houston, Houston, Texas, United States of America; 8 School of Public Health, University of Minnesota, Minneapolis, Minnesota, United States of America; 9 Jackson State University, Jackson, Mississippi, United States of America; 10 Medical Genetics Institute, Cedars-Sinai Medical Center, Los Angeles, California, United States of America; 11 University of Virginia School of Medicine, Charlottesville, Virginia, United States of America; 12 University Hospitals, Case Medical Center and Case Western Reserve University, Cleveland, Ohio, United States of America; 13 University of Washington, Seattle, Washington, United States of America; 14 Group Health Research Institute, Group Health Cooperative, Seattle, Washington, United States of America; 15 National Heart, Lung, and Blood Institute, National Institutes of Health, Bethesda, Maryland, United States of America; 16 JM-United States Department of Agriculture Human Nutrition Research Center on Aging at Tufts University, Boston, Massachusetts, United States of America; 17 Northwestern University Feinberg School of Medicine, Chicago, Illinois, United States of America; 18 Children’s Hospital Oakland Research Institute, Oakland, California, United States of America; 19 Boston University, Boston, Massachusetts, United States of America; 20 The National Heart, Lung, Blood Institute’s Framingham Heart Study, Framingham, Massachusetts, United States of America; 21 Baylor College of Medicine, Houston, Texas, United States of America; 22 University of Pennsylvania School of Medicine, Philadelphia, Pennsylvania, United States of America; University of Tor Vergata, Italy

## Abstract

**Background:**

Whereas it is well established that plasma lipid levels have substantial heritability within populations, it remains unclear how many of the genetic determinants reported in previous studies (largely performed in European American cohorts) are relevant in different ethnicities.

**Methodology/Principal Findings:**

We tested a set of ∼50,000 polymorphisms from ∼2,000 candidate genes and genetic loci from genome-wide association studies (GWAS) for association with low-density lipoprotein cholesterol (LDL-C), high-density lipoprotein cholesterol (HDL-C), and triglycerides (TG) in 25,000 European Americans and 9,000 African Americans in the National Heart, Lung, and Blood Institute (NHLBI) Candidate Gene Association Resource (CARe). We replicated associations for a number of genes in one or both ethnicities and identified a novel lipid-associated variant in a locus harboring *ICAM1*. We compared the architecture of genetic loci associated with lipids in both African Americans and European Americans and found that the same genes were relevant across ethnic groups but the specific associated variants at each gene often differed.

**Conclusions/Significance:**

We identify or provide further evidence for a number of genetic determinants of plasma lipid levels through population association studies. In many loci the determinants appear to differ substantially between African Americans and European Americans.

## Introduction

Plasma concentrations of lipids and lipoproteins [low-density lipoprotein cholesterol (LDL), high-density lipoprotein cholesterol (HDL-C), triglycerides (TG)] are heritable risk factors for cardiovascular disease [1], [2]. Recently, genome-wide association mapping of common variants (>5% minor allele frequency) in individuals of European ancestry has proven useful in identifying genetic loci contributing to plasma lipids [3]. Roughly one third of these loci harbor genes with previously recognized involvement in lipid metabolism, many by virtue of having rare variants that result in Mendelian disorders. The other loci are suspected to harbor novel lipid regulators, the study of which has the potential of providing important new insights into lipid biology.

As promising as these observations may be, several critical questions remain – How many of the previously reported loci are genuine causal determinants of plasma lipid levels? Are there more loci associated with plasma lipids that are discoverable with techniques besides genome-wide association mapping? Do any of these loci confer effects on lipids in an ethnic-specific manner? To address these questions, we utilized the National Heart, Lung, and Blood Institute (NHLBI) Candidate Gene Association Resource (CARe) comprising more than 40,000 individuals from nine prospective cohorts with measured lipid phenotypes and genotype information obtained using the “ITMAT-Broad-CARe” array, or IBC array, with ∼50,000 polymorphisms from ∼2,000 candidate genes/loci [4], [5]. Most of the polymorphisms on this array were obtained from a systematic literature search for candidate genes implicated in cardiovascular diseases; these were supplemented with polymorphisms from loci identified in early genome-wide association studies (GWAS) for lipids [5].

We initiated this study with specific hypotheses: (1) Common DNA sequence variants in previously reported genes and loci, as well as additional novel loci, are associated with plasma lipids; (2) At some of these loci, the specific variants related to plasma lipids differ between ethnic groups. To test these hypotheses, we performed association analyses of the polymorphisms on the IBC array in 25,000 European Americans and 9,000 African Americans in CARe.

## Materials and Methods

### Ethics Statement

All participants in each of the CARe cohorts gave informed written consent. The Institutional Review Boards (IRBs) of each CARe cohort (i.e., the IRBs for each cohort’s field centers, coordinating center, and laboratory center) have reviewed and approved the cohort’s interaction with CARe. The study described in this manuscript was approved by the Committee on the Use of Humans as Experimental Subjects (COUHES) of the Massachusetts Institute of Technology.

### CARe Study Design and Quality Control

A full description of the CARe Study is found elsewhere [4]. In brief, DNA samples and phenotypic information from nine NHLBI prospective cohorts [the Atherosclerosis Risk In Communities (ARIC) study, the Coronary Artery Risk Development In Young Adults (CARDIA) study, the Cleveland Family Study (CFS), the Cardiovascular Health Study (CHS), the Cooperative Study of Sickle Cell Disease (CSSCD), the Framingham Heart Study (FHS), the Jackson Heart Study (JHS), the Multi-Ethnic Study of Atherosclerosis (MESA), and the Sleep Heart Health Study (SHHS)] were assembled at the Broad Institute of MIT and Harvard. We used the following strata of cohort and ethnicity (EA = European American, AA = African American): ARIC-AA, CARDIA-AA, CFS-AA, CHS-AA, JHS, MESA-AA, ARIC-EA, CARDIA-EA, CFS-EA, CHS-EA, FHS, MESA-EA. Plasma lipid levels–LDL-C, HDL-C, TG–from the baseline examinations of cohort participants were used (Table S1). All phenotypes, including plasma lipid levels, had been standardized according to the Clinical Data Interchange Standard Consortium Study Data Tabulation Model (CDISC SDTM).

All DNA samples passing initial quality checks were interrogated with the IBC v2 chip for genotyping of 49,320 total single nucleotide polymorphisms (SNPs). Samples with overall genotyping success rate <95%, individual SNPs with genotyping success rate <95% within a cohort, monomorphic SNPs, and SNPs mapping to multiple genomic loci were removed. An inbreeding coefficient was calculated for each sample as a measure of heterozygosity, with those samples exceeding 4 standard deviations from the mean (suggesting poor DNA quality if too low, or sample contamination if too high) being removed. In cases of identical DNA samples, the sample with the lowest genotyping success rate was removed. Samples that shared 5% or more of their genome with many other samples were also removed. Additional outlier samples as determined by multidimensional scaling were also removed. SNPs for which genotype missingness could be predicted by surrounding haplotypes were removed, as were any SNPs found to be associated with chemistry plates. Pedigrees and SNPs not conforming to Mendelian expectations were identified and, where appropriate, samples were removed. These analyses were all performed with PLINK [6].

Because different ethnic groups were represented, with the expectation of differing genotype frequencies and admixture, no filters were applied for minor allele frequency or Hardy-Weinberg *P* values. The Hardy-Weinberg *P* value for each index SNP in each cohort is listed in Table S2.

About 2,000 ancestry-informative markers were included in the IBC v2 chip [4]. ANCESTRYMAP [7] was used to generate estimates for global ancestry in African American samples. EIGENSTRAT [8] was used for self-reported African Americans and European Americans separately to generate ten principal components each accounting for population stratification. We found that the first principal component for African Americans was highly correlated with the global ancestry estimates (*r* ^2^>0.98).

### Phenotype Modeling

We modeled the lipid phenotypes in the following ways. LDL-C was calculated according to Friedewald’s formula: LDL-C = total cholesterol – HDL-C – (TG ÷ 5). If a TG value was >400 mg/dL, LDL-C was treated as a missing value. For individuals on lipid-lowering therapy, the LDL-C value was multiplied by 1.42 to model a 30% reduction in LDL-C on therapy. This represents the average expected reduction in LDL-C with a first-generation statin, the most commonly used lipid-lowering medication during the study periods of most of the cohorts [9]. TG values were log(10)-transformed. Sex-specific phenotype residuals were constructed within strata of cohort and ethnicity after accounting for age and age^2^. Each set of residuals was standardized to a mean of zero and a standard deviation of one. The standardized residual served as the phenotype in genotype-phenotype association analyses. Generation of residuals was performed with the R statistical package (The R Foundation for Statistical Computing, Vienna, Austria).

### Association Testing

For each of the three traits, we used linear regression to test SNP-phenotype associations in each stratum of cohort and ethnicity assuming an additive genetic model, using the ten calculated principal components as covariates: phenotype ∼ genotype + PC1+ PC2+ PC3+ PC4+ PC5+ PC6+ PC7+ PC8+ PC9+ PC10. These association analyses were performed in PLINK. Genotype-phenotype associations within each ethnic group were assessed by weighted z-score-based fixed-effects meta-analysis and effect size estimates (β values) generated by inverse-variance weighted meta-analysis, both using METAL (G. Abecasis, University of Michigan). Genomic control correction was applied to each cohort individually prior to meta-analysis, and a final genomic control correction was applied to each ethnicity’s meta-analysis dataset. For all cross-ethnic comparisons, only SNPs available in all cohorts (12 total) were considered. We considered *P* = 1×10^−6^ to be the threshold for statistical significance, applying a Bonferonni correction for the number of SNPs tested (0.05 ÷ 50,000).

We performed sensitivity analyses for two cohorts for which there were significant numbers of related individuals–CFS and FHS–to account for family relationships, using a linear mixed effects (LME) model to analyze the lipid residuals, with the SNP genotype treated as a fixed effect, and a random effect according to the degree of relatedness within a family [10]. Upon substitution of these data into the meta-analyses for each lipid trait, there were essentially no differences in the SNPs found to be statistically significant, and these data were not considered further.

To identify SNPs exerting sex-specific effects, we added sex as a covariate in the above linear regression model and tested for a formal SNP×sex interaction; the results were meta-analyzed as described above. Similarly, to test for SNP×SNP interactions among the most strongly associated SNPs at each of the identified loci, we added the minor allele count for each SNP as a covariate, in turn, into the above model (with sex removed), testing for a formal interaction between each SNP pair combination.

For those loci containing IBC-significant SNPs in both ethnicities, we performed SNP conditional analyses. These were conducted by adding SNP allele counts as extra covariates to the initial linear regression model described above. The conditional analyses were performed iteratively, with additional independent SNPs identified at each step in each locus added to the model. Adjusted *R* ^2^ values for each SNP included in the conditional analyses, as well as for the combination of independently significant SNPs at each locus, were calculated by replicating the relevant linear regression model in the largest cohort (ARIC for both ethnicities) using Statistics Package for Social Sciences (SPSS) software (version 16.0; SPSS Inc., Chicago, IL), with principal components excluded.

## Results

### Replication of Reported Lipid-associated Loci and Identification of New Loci

Nineteen lipid-associated loci that were reported in an early set of lipid GWAS studies of individuals of European ancestry were included in the IBC v2 array–these loci harbor the genes *SORT1*, *CILP2/PBX4*, *GALNT2*, *MLXIPL*, *TRIB1*, *ANGPTL3*, *APOB*, *APOE*, *HMGCR*, *LDLR*, *PCSK9*, *ABCA1*, *APOA1/APOA5*, *CETP*, *LIPC*, *LIPG*, *LPL*, *GCKR*, and *MVK-MMAB* [11]–[14]. We found that SNPs at all 19 loci replicated in the CARe European American cohorts (Table 1) at the pre-specified IBC significance level (*P*<1×10^−6^). Importantly, there was no overlap between the GWAS cohorts and the CARe cohorts. We also found that SNPs at 10 loci replicated in the CARe African American cohorts–*SORT1* (sortilin), *APOB* (apolipoprotein B), *APOE* (apolipoprotein E), *LDLR* (LDL receptor), *PCSK9* (proprotein convertase subtilisin/kexin type 9), *ABCA1* [ATP-binding cassette, sub-family A (ABC1), member 1], *APOA1* (apolipoprotein A-I), *CETP*, (cholesteryl ester transfer protein), *LIPC* (hepatic lipase), and *LPL* (lipoprotein lipase) (Table 1). All of these genes, with the exception of *SORT1*, have well-described functions in lipoprotein metabolism.

**Table 1:** SNP variants associated with lipid traits in CARe European Americans and African Americans.

Gene(s) of interest in associated interval	Trait	SNP	Chr	Position	Alleles	European American MAF	African American MAF	European American *P*	African American *P*	European American β	African American β	Direction (EA, AA)
*PCSK9*	LDL	rs11591147	1	55278235	T, G	0.016	0.0033	**1.74×10^−28^**	0.00011	–0.44	–0.51	– –
		rs11806638	1	55290748	A, C	0.056	0.33	0.012	**5.05×10^−11^**	–0.054	–0.11	– –
*ANGPTL3*	TG	rs1748197	1	62828700	A, G	0.34	0.34	**6.23×10^−7^**	0.012	–0.053	–0.042	– –
*SORT1*	LDL	rs7528419	1	109618715	A, G	0.22	0.28	**1.69×10^−51^**	1.34×10^−16^	0.18	0.15	+ +
		rs12740374	1	109619113	T, G	0.22	0.26	2.90×10^−51^	**9.33×10^−20^**	–0.18	–0.17	– –
*GALNT2*	HDL	rs4846918	1	228367209	T, C	0.15	0.14	**4.22×10^−7^**	0.45	0.069	0.016	+ +
*APOB*	LDL	rs934197	2	21120966	A, G	0.32	0.12	**2.88×10^−34^**	0.0081	0.13	0.065	+ +
		rs562338	2	21141826	A, G	0.18	0.39	7.60×10^−34^	**2.66×10^−8^**	–0.15	–0.092	– –
	HDL	rs673548	2	21091049	A, G	0.21	0.22	**6.45×10^−9^**	0.044	0.068	0.037	+ +
	TG	rs1042034	2	21078786	T, C	0.21	0.16	**2.23×10^−10^**	0.00031	0.068	0.079	+ +
*GCKR*	TG	rs1260326	2	27584444	T, C	0.41	0.14	**6.79×10^−35^**	8.23×10^−5^	0.037	0.088	+ +
*ABCG5-ABCG8*	LDL	rs4953023	2	43927504	A, G	0.070	0.078	**2.41×10^−8^**	0.0011	–0.11	–0.10	– –
*HMGCR*	LDL	rs12916	5	74692295	T, C	0.41	0.25	**5.50×10^−13^**	0.099	–0.075	–0.031	– –
*LPA*	LDL	rs10455872	6	160930108	A, G	0.067	0.010	**2.81×10^−12^**	0.24	–0.14	–0.081	– –
*NPC1L1*	LDL	rs17725246	7	44548511	T, C	0.19	0.25	**5.56×10^−7^**	0.025	–0.061	–0.041	– –
*MLXIPL*	TG	rs17145750	7	72664314	T, C	0.15	0.11	**4.22×10^−25^**	0.12	–0.0017	–0.040	– –
*CD36*	HDL	rs3211938	7	80138385	T, G	0.00010	0.083	0.030	**2.60×10^−12^**	–1.31	–0.1931	– –
*LPL*	HDL	rs3916027	8	19869148	A, G	0.26	0.41	**1.01×10^−31^**	1.93×10^−7^	0.13	0.082	+ +
		rs13702	8	19868772	T, C	0.29	0.48	2.14×10^−28^	**3.12×10^−11^**	–0.12	–0.10	– –
	TG	rs3916027	8	19869148	A, G	0.26	0.41	**1.21×10^−40^**	2.65×10^−8^	–0.0072	–0.089	– –
		rs327	8	19863816	T, G	0.27	0.41	2.72×10^−36^	**1.03×10^−9^**	0.0026	0.097	+ +
*TRIB1*	LDL	rs6982636	8	126548497	A, G	0.47	0.36	**4.29×10^−9^**	1.0	–0.059	–0.00	– –
	HDL	rs2980880	8	126550154	A, G	0.31	0.32	**5.85×10^−7^**	0.46	0.052	0.012	+ +
	TG	rs2980875	8	126550929	A, G	0.47	0.37	**1.15×10^−15^**	0.19	0.057	0.023	+ +
*ABCA1*	HDL	rs1883025	9	106704122	T, C	0.26	0.34	**6.25×10^−7^**	0.031	–0.055	–0.036	– –
		rs2515629	9	106634185	A, G	0.17	0.17	0.53	**2.04×10^−7^**	0.0079	0.11	+ +
*FADS1-FADS2-FADS3*	HDL	rs1535	11	61354548	A, G	0.33	0.15	**5.29×10^−9^**	0.00055	0.059	0.074	+ +
*APOA1-C3-A4-A5*	HDL	rs10750097	11	116169250	A, G	0.21	0.43	**2.76×10^−18^**	0.55	0.10	–0.0094	+ –
	TG	rs2075290	11	116158506	T, C	0.070	0.055	**8.13×10^−51^**	0.00044	–0.064	–0.12	– –
		rs9804646	11	116170289	T, C	0.079	0.36	0.011	**4.81×10^−7^**	–0.039	–0.083	– –
*MMAB-MVK*	HDL	rs2075440	12	108343483	A, G	0.45	0.35	**6.78×10^−7^**	0.45	–0.048	–0.013	– –
*LIPC*	HDL	rs2070895	15	56511231	A, G	0.21	0.47	**3.90×10^−24^**	**3.93×10^−10^**	0.12	0.097	+ +
*CETP*	HDL	rs17231506	16	55552029	T, C	0.32	0.14	**1.50×10^−120^**	1.54×10^−8^	0.24	0.12	+ +
		rs17231520	16	55553328	A, G	–	0.070	–	**7.65×10^−58^**	–	0.50	0+
*LCAT*	HDL	rs2107369	16	66673519	T, G	0.14	0.26	**9.23×10^−8^**	0.58	–0.076	–0.010	– –
		rs255052	16	66582496	A, G	0.15	0.22	6.63×10^−7^	**1.22×10^−8^**	0.069	0.11	+ +
*HPR*	LDL	rs2000999	16	70665594	A, G	0.20	–	**2.39×10^−8^**	–	0.069	–	+0
*LIPG*	HDL	rs1943981	18	45423813	A, T	0.16	0.05	**1.83×10^−13^**	0.41	–0.095	–0.027	– –
*ANGPTL4*	HDL	rs2278236	19	8337581	A, G	0.48	0.47	**6.46×10^−7^**	0.96	0.048	–0.00060	+ –
*ICAM1*	LDL	rs5030359	19	10249462	A, G	–	0.0086	–	**1.24×10^−8^**	–	–0.52	0–
*LDLR*	LDL	rs6511720	19	11063306	T, G	0.12	0.14	**1.38×10^−49^**	**1.75×10^−18^**	–0.23	–0.20	– –
*CSPG3-CILP2-PBX4*	TG	rs3794991	19	19471596	T, C	0.090	0.066	**4.83×10^−10^**	0.98	–0.084	0.0012	– +
*APOE*	LDL	rs12721046	19	50113094	A, G	0.15	0.030	**2.24×10^−29^**	0.00098	0.16	0.16	+ +
		rs389261	19	50112183	A, G	–	0.25	–	**1.10×10^−14^**	–	0.15	0+
	HDL	rs12721046	19	50113094	A, G	0.15	0.030	**1.67×10^−7^**	0.056	–0.075	–0.087	– –
	TG	rs439401	19	50106291	T, C	0.37	0.14	**1.85×10^−15^**	0.88	–0.022	–0.0046	– –
		rs12721054	19	50114427	A, G	0.00047	0.12	0.60	**5.55×10^−26^**	0.33	0.26	+ +
*PLTP*	HDL	rs4810479	20	43978455	T, C	0.25	0.40	**1.90×10^−9^**	0.00013	0.067	0.062	+ +

AA = African American; EA = European American; MAF = major allele frequency. Minor allele frequencies all derived from ARIC (largest cohort for both African Americans and European Americans). Bold indicates the most highly associated SNP (lowest *P* value) at IBC significance (*P*<1×10^−6^) for locus + trait + ethnicity. Direction is modeled on the first allele listed in Alleles column.

We also found significant associations for a number of IBC SNPs chosen for their proximity to candidate genes selected from the literature. Of these, eight loci–*FADS1* (fatty acid desaturase 1), *PLTP* (phospholipid transfer protein), *LCAT* (lecithin-cholesterol acyltransferase), *ANGPTL4* (angiopoietin-like 4), *ABCG5/ABCG8* (ATP-binding cassette sub-family G members 5 and 8; sterolin-1 and -2), *LPA* [lipoprotein(a)], *NPC1L1* [NPC1 (Niemann-Pick disease, type C1, gene)-like 1], *HPR* (haptoglobin-related protein)–were shown to have genome-wide-significant lipid-associated SNP variants in GWAS reported subsequent to the fabrication of the IBC array (Table 1) [3]. All of these genes, with the exception of *FADS1* and *HPR*, have well-described functions in lipoprotein metabolism. The *LCAT* locus was notable for being IBC-significant for HDL-C in both CARe European Americans and African Americans. An additional significant locus in African Americans harboring *CD36* (cluster of differentiation 36; thrombospondin receptor) had previously been reported in a candidate gene study; indeed, the same SNP that was IBC-significant (rs3211938) was shown in that prior study to be a nonsense coding variant that resulted in CD36 deficiency in a homozygous individual and was associated with increased HDL-C levels (*P* = 0.00018) and decreased TG levels (*P* = 0.0059) in an African American cohort [15].

Finally, one locus harbors an IBC-significant variant that has not previously been reported to be associated with lipid traits–*ICAM1* (intercellular adhesion molecule 1) in CARe African Americans (Table 1). Of note, the *P* value of association (1.24×10^−8^) for the *ICAM1* SNP rs5030359 is not only IBC-significant but also genome-wide-significant. This variant is of low frequency in African Americans (just under 1%) and is virtually absent in European Americans. Interestingly, this variant demonstrated the largest effect size for any trait + ethnicity combination (β = –0.52).

### Architecture of Lipid Loci in African Americans Compared to European Americans

Comparing each index SNP in each of the lipid loci in European Americans to African Americans, we noted quite varied patterns of association. In some cases, the effect size estimates and minor allele frequencies (MAFs) are quite similar in two ethnicities. An example is SNP rs6511720 in the *LDLR* locus, with β = –0.23 (in standard deviation units) and MAF = 0.12 in European Americans, β = –0.20 and MAF = 0.14 in African Americans for LDL-C (Table 1). The relative *P* values (*P* = 1.38×10^−49^ vs. *P* = 1.75×10^−18^) can be attributed solely to the different sample sizes (25,000 European Americans vs. 9,000 African Americans). Another example is rs1748197 in the *ANGPTL3* (angiopoietin-like 3) locus, which has β = –0.053 and MAF = 0.34 in European Americans, β = –0.042 and MAF = 0.34 in African Americans for TG (*P* = 6.23×10^−7^ vs. *P* = 0.012).

Other index SNPs diverged considerably in the two ethnicities. Some of the SNPs displayed considerable differences in their estimated effect sizes despite similar MAFs. Notable examples include rs2278236 in the *ANGPTL4* locus, with β = 0.048 (MAF = 0.48; *P* = 6.46×10^−7^) in European Americans and β = –0.00060 (MAF = 0.48; *P* = 0.96) in African Americans; rs4846918 in the *GALNT2* (UDP-N-acetyl-alpha-D-galactosamine:polypeptide N-acetylgalactosaminyltransferase 2) locus, with β = 0.069 (MAF = 0.15; *P* = 4.22×10^−7^) in European Americans and β = 0.016 (MAF = 0.14; *P* = 0.45) in African Americans for HDL-C; and rs2515629 in the *ABCA1* locus, with β = 0.0079 (MAF = 0.17; *P* = 0.53) in European Americans and β = 0.11 (MAF = 0.17; *P* = 2.04×10^−7^) in African Americans for HDL-C. These findings indicate considerable heterogeneity in the architecture of lipid-related loci in the two ethnicities.

We took advantage of the dense genotyping in each locus on the IBC v2 array to perform more detailed comparisons of the 11 loci that harbored IBC-significant SNPs for both ethnicities (Table 2). In only two of the loci (*LIPC* for HDL-C, *LDLR* for LDL-C) was the same SNP the most highly associated for the trait in each ethnicity. At an additional four loci (*SORT1* with LDL-C, *APOB* with LDL-C, *LPL* with HDL-C and TG, *LCAT* with HDL), the most highly associated SNP in one ethnicity was among the most highly associated SNPs in the other ethnicities. In the remaining five loci (*PCSK9* for LDL-C, *ABCA1* for HDL-C, *APOA1-C3-A4-A5* for TG, *CETP* for HDL, *APOE* for LDL-C and TG), the most highly associated SNPs in the ethnicities did not overlap.

**Table 2:** Independent SNP variants associated with lipid traits within single gene regions.

Gene(s) of interest in associated interval	Trait	SNP	Chr	Position	Alleles	European American MAF	African American MAF	European American *P*	African American *P*	Direction (EA, AA)	European American SNP *R* ^2^	African American SNP *R* ^2^	European American Locus *R* ^2^	African American Locus *R* ^2^
*PCSK9*	LDL	rs11591147	1	55278235	T, G	0.016	0.0033	1.73×10^−28^ *	0.00025	– –	0.007	0.005	0.011	0.003
		rs11806638	1	55290748	A, C	0.056	0.33	0.012	4.65×10^−11^ *	– –	0.000	0.002		
		rs499883	1	55291762	A, G	0.40	–	1.45×10^−11^ **	–	+0	0.004	–		
		rs505151	1	55301775	A, G	–	0.2457	–	9.88×10^−10^ **	0–	–	0.002		
*SORT1*	LDL	rs7528419	1	109618715	A, G	0.22	0.28	1.69×10^−51^ *	4.98×10^−17^	+ +	0.013	0.005	0.013	0.006
		rs12740374	1	109619113	T, G	0.22	0.26	2.89×10^−51^	2.32×10^−20^ *	– –	0.013	0.006		
*APOB*	LDL	rs5742904	2	21082665	T, C	0.00068	–	7.53×10^−14^ ***	–	+0	0.004	–	0.016	0.004
		rs934197	2	21120966	A, G	0.32	0.12	2.88×10^−34^ *	0.0076	+ +	0.007	0.001		
		rs562338	2	21141826	A, G	0.18	0.39	7.60×10^−34^ **	1.08×10^−8^ *	– –	0.008	0.004		
*LPL*	HDL	rs268	8	19857809	A, G	0.020	0.0031	4.55×10^−11^ **	0.040	+ +	0.003	0.000	0.009	0.006
		rs13702	8	19868772	T, C	0.29	0.48	2.14×10^−28^	1.43×10^−10^ *	– –	0.005	0.006		
		rs3916027	8	19869148	A, G	0.26	0.41	1.01×10^−31^ *	7.99×10^−7^	+ +	0.006	0.003		
		rs10503669	8	19891970	A, C	0.098	0.057	3.77×10^−28^ ***	0.00084	+ +	0.005	0.001		
*LPL*	TG	rs268	8	19857809	A, G	0.020	0.0031	9.49×10^−13^ **	0.5053	– –	0.003	0.000	0.012	0.004
		rs327	8	19863816	T, G	0.27	0.41	2.72×10^−36^	1.03×10^−9^ *	+ +	0.008	0.004		
		rs12679834	8	19864713	T, C	0.10	0.082	1.29×10^−32^ ***	3.65×10^−8^	++	0.006	0.004		
		rs3916027	8	19869148	A, G	0.26	0.41	1.20×10^−40^ *	2.65×10^−8^	– –	0.009	0.004		
*ABCA1*	HDL	rs2515629	9	106634185	A, G	0.17	0.17	0.53	2.98×10^−7^ *	+ +	0.000	0.004	0.002	0.004
		rs2472449	9	106644018	T, G	0.34	0.22	1.40×10^−6^ **	9.50×10^−5^	+ +	0.001	0.002		
		rs1883025	9	106704122	T, C	0.26	0.34	6.25×10^−7^ *	0.031	– –	0.001	0.000		
*APOA1-C3-A4-A5*	TG	rs180327	11	116128869	T, C	0.37	0.49	3.44×10^−23^	1.79×10^−5^ **	– –	0.008	0.000	0.025	0.003
		rs2075290	11	116158506	T, C	0.070	0.055	8.14×10^−51^ *	0.00044	– –	0.013	0.000		
		rs12287066	11	116167541	T, G	0.068	0.17	3.54×10^−34^ **	0.031	+ +	0.010	0.000		
		rs9804646	11	116170289	T, C	0.079	0.36	0.011	4.81×10^−7^ *	– –	0.001	0.003		
*LIPC*	HDL	rs4775041	15	56461987	C, G	0.29	0.13	2.69×10^−20^ **	0.21	+ +	0.004	0.000	0.011	0.005
		rs2070895	15	56511231	A, G	0.21	0.47	3.91×10^−24^ *	1.35×10^−10^ *	+ +	0.007	0.005		
*CETP*	HDL	rs17231506	16	55552029	T, C	0.32	0.14	1.50×10^−120^ *	2.41×10^−8^	+ +	0.025	0.005	0.036	0.050
		rs4783961	16	55552395	A, G	0.499	0.43	3.58×10^−31^	1.17×10^−43^ **	+ +	0.006	0.026		
		rs17231520	16	55553328	A, G	–	0.070	–	2.46×10^−56^ *	0+	–	0.037		
		rs7499892	16	55564091	T, C	0.17	0.39	4.20×10^−78^	1.04×10^−26^ ***	– –	0.017	0.012		
		rs5883	16	55564854	T, C	0.058	0.11	2.66×10^−8^ ***	1.72×10^−12^	+ +	0.000	0.006		
		rs11076176	16	55564947	T, G	0.17	–	8.88×10^−67^ **	–	+0	0.013	–		
		rs5880	16	55572592	C, G	0.050	0.0096	7.28×10^−33^ ****	0.0044	– –	0.007	0.001		
*LCAT*	HDL	rs35673026	16	66534352	T, C	–	0.0038	–	1.54×10^−6^ **	0+	–	0.004	0.002	0.011
		rs255052	16	66582496	A, G	0.15	0.22	6.64×10^−7^	5.02×10^−9^ *	+ +	0.002	0.007		
		rs2107369	16	66673519	T, G	0.14	0.26	9.23×10^−8^ *	0.70	– –	0.002	0.000		
*LDLR*	LDL	rs6511720	19	11063306	T, G	0.12	0.14	1.39×10^−49^ *	2.29×10^−18^ *	– –	0.008	0.009	0.010	0.012
		rs17242787	19	11063460	A, T	–	0.019	–	2.19×10^−9^ **	0–	–	0.003		
		rs688	19	11088602	T, C	0.43	0.10	1.66×10^−11^ **	0.0014	+ +	0.002	0.003		
*APOE*	LDL	rs8106922	19	50093506	A, G	0.39	0.24	7.25×10^−5^	5.75×10^−6^ ***	– –	0.000	0.003	0.010	0.019
		rs405509	19	50100676	T, G	0.49	0.26	1.93×10^−11^	1.89×10^−12^ **	+ +	0.002	0.005		
		rs769450	19	50102284	A, G	0.40	0.37	6.93×10^−7^ ***	0.00015	+ +	0.001	0.003		
		rs389261	19	50112183	A, G	–	0.25	–	2.72×10^−14^ *	0+	–	0.003		
		rs12721046	19	50113094	A, G	0.15	0.030	2.23×10^−29^ *	0.0017	+ +	0.006	0.003		
		rs12721109	19	50139061	A, G	0.022	0.0028	7.72×10^−27^ **	0.023	– –	0.004	0.000		
*APOE*	TG	rs439401	19	50106291	T, C	0.37	0.14	1.85×10^−15^ *	0.88	– –	0.003	0.000	0.003	0.012
		rs12721054	19	50114427	A, G	0.00047	0.12	0.60	3.73×10^−25^ *	+ +	0.000	0.012		

AA = African American; EA = European American; MAF = major allele frequency. Minor allele frequencies and R^2^ values all derived from ARIC (largest cohort for both African Americans and European Americans). Direction is modeled on the first allele listed in “Alleles” column. Independent SNPs (when they exist) are indicated as: * = lowest *P* value for locus + trait + ethnicity; ** = lowest *P* value at or near IBC significance (*P*<1×10^−6^) after conditioning on first SNP; *** = lowest *P* value at or near IBC significance (*P*<1×10^−6^) after conditioning on first two independent SNPs; etc. Locus R2 values calculated from all independently significant SNPs at the corresponding locus.

For each ethnicity we performed conditional analyses with the most highly associated SNPs in each of the 11 loci to uncover any additional, independently associated SNPs (at IBC significance) in the same gene regions (Table 2). We performed this iteratively until no association signals remained in each locus. By this criterion, we identified up to four independent SNP variants (in the case of *CETP* with HDL-C in European Americans) per locus. Remarkably, only one locus (*SORT1*) harbored a single independent SNP in each ethnic group.

For many gene regions, the independent SNPs did not colocalize in the two ethnicities. For example, in the *PCSK9* locus, the most highly associated SNPs (rs11591147 in European Americans and rs11806638 in African Americans) are more than 10 kb apart and show much weaker associations (and are much less common) in the other ethnicity (Table 2). Similarly, the second independent SNPs (rs499883 in European Americans and rs505151 in African Americans) are more than 10 kb apart and are each absent in the other ethnicity. Similar patterns are seen with *APOB*, *APOA1-C3-A4-A5*, and *LCAT*. More complex patterns are seen in loci such as *LPL*, *CETP*, and *APOE*, where some independent SNPs colocalize (within a few kb of each other) and others are distant.

Notably, for two loci/trait combinations–*LCAT* with HDL, *APOE* with TG–the strength of association of SNPs is greater in African Americans than in European Americans (Table 2). For *APOE*, this is due to the rarity of the index SNP (rs12721054) in European Americans (MAF = 0.00047 vs. MAF = 0.12), since the effect size estimates are similar in the two ethnic groups (β = 0.33 in European Americans vs. β = 0.26 in African Americans). For *LCAT*, rarity accounts for the second independent SNP in African Americans (rs35673026, absent in European Americans) but not the most highly associated SNP (rs255052, similarly common in European Americans, MAF = 0.15 vs. MAF = 0.22, with modestly different effect size estimates, β = 0.069 vs. β = 0.11). No second independent SNP for *LCAT* was identified in European Americans.

Table 2 also reports the proportion of the variance (adjusted *R* ^2^) in the respective traits explained by each SNP and the combination of the independently significant SNPs at each locus. The greatest proportion of variance explained by a single SNP in African Americans was 3.7% (rs17231520 for HDL-C); for European Americans it was 2.5% (rs17231506 for HDL-C). These SNPs are both located in the *CETP* locus on chromosome 16. Unsurprisingly, the *CETP* locus explained the greatest proportion of variance, in any trait, for both African Americans and European Americans (explaining 5.0% and 3.6% of the variance in HDL-C, respectively). For African Americans, the combination of all independently significant SNPs at the 11 loci that harbored IBC-significant SNPs for both ethnicities explained 4.5%, 7.7%, and 1.9% of the variance in LDL-C, HDL-C, and TG, respectively. For European Americans, 6.2%, 6.0%, and 4.1% of the variance in LDL-C, HDL-C, and TG were explained, respectively.

### SNP×SNP Interactions and SNPs Exerting Sex-specific Effects

We investigated whether any SNP×SNP interactions existed between any of the most significant SNPs identified at each of the 19 above-mentioned loci for their respective traits; none were identified (Tables S3, S4, S5, S6, S7, S8). The lowest *P* value (0.002) was generated for the interaction between SNPs rs10455872 (*LPA*) and rs934197 (*APOB*) for LDL-C in African Americans, but was far from IBC-significant and withstands a Bonferroni correction of only 25 tests (there were 120 SNP×SNP interactions tested for LDL-C among African Americans).

Similarly, no IBC-significant SNP×sex interactions were observed among the SNPs identified in Table 1 (Tables S9, S10, S11). Of note, the SNP rs4810479 (*PLTP*) generated a *P* value of 1.51×10^−4^ for HDL-C among European Americans and was highly significant among women (*P* = 7.11×10^−12^) but not men (*P* = 0.12). The *P* value of this interaction was two orders of magnitude smaller than for any of the other SNPs for any lipid trait, with the exception of rs439401 (*P* = 1.30×10^−3^ for TG among European Americans). Expanding the search to all SNPs on the IBC chip failed to identify any further IBC-significant SNP×sex interactions (Tables S12, S13).

## Discussion

In this work, we identify or provide further evidence for a number of genetic determinants of plasma lipid levels through population association studies of two ethnicities. Specifically, the results of these studies support each of our hypotheses.

### Common DNA Sequence Variants in Previously Reported Genes and Loci, as well as Additional Novel Loci, are Associated with Plasma Lipids

We found that all 19 lipid-associated loci identified in early GWAS studies replicated in the combined CARe European American cohorts (∼25,000 individuals), and 10 of the loci replicated in the combined CARe African American cohorts (∼9,000 individuals). It is likely that with genotyping in additional African American individuals, increased power will ultimately allow replication of many of the remaining nine loci. We identified an additional 10 loci associated with one or more lipid traits at Bonferroni-corrected statistical significance. Eight of these loci were mapped in GWAS studies subsequent to the design of the IBC array, and one locus had been identified in a prior non-GWAS study. The remaining locus, *ICAM1*, is novel. The identification of *ICAM1* is of particular interest because its association with LDL-C was exclusive to African Americans.

These findings give a high degree of confidence that most, if not all, of the reported GWAS lipid loci harbor authentic determinants of plasma lipid levels deserving of further functional investigation, and that many of the loci are relevant not just in European American populations but more generally in global populations.

### At Some Lipid-associated Loci, the Specific Variants Related to Plasma Lipids Differ between Ethnic Groups

We were able to use the dense SNP genotyping in loci available via the IBC array to analyze lipid-associated loci in an unprecedented level of detail, particularly in African Americans. Our analyses demonstrate that at many loci there are major differences in genetic architecture between European Americans and African Americans. These differences manifest in at least three ways.

First, some of the most highly associated SNPs in one ethnicity are rare or absent in the other ethnicity. This is a well-established phenomenon; for example, truncation mutations in *PCSK9* that are of low frequency in African Americans, but absent in European Americans, have been shown to result in a robust reduction in LDL-C levels and coronary heart disease risk [16], [17]. Many of the discrepancies in lipid associations of SNPs between the two ethnicities can be attributed primarily to differences in allele frequency. For example, rs3211938 in *CD36* is much more highly associated with HDL-C in African Americans (*P* = 2.60×10^−12^) than in European Americans (*P* = 0.030), with a large discrepancy in MAFs (8.3% vs. 0.01%) (Table 1). Similarly, rs12721054 in *APOE* is both more highly associated with TG and more frequent in African Americans (*P* = 5.55×10^−25^, MAF = 12%) than in European Americans (*P* = 0.60, MAF = 0.047%). These variants may represent causal variants, as is the case with the nonsense coding variant rs3211938 in *CD36* [15].

Second, some of the most highly associated SNPs in one ethnicity are poorly associated in the other ethnicity despite similar MAFs in both groups. An example is rs2515629 in the *ABCA1* locus (*P* = 2.04×10^−7^, MAF = 0.17, β = 0.11 in African Americans; *P* = 0.53, MAF = 0.17, β = 0.0079 in European Americans) (Table 2). In cases like this, it seems unlikely that the SNP represents a causal variant that acts exclusively in one ethnic group. Instead, rs2515629 is likely to be in strong linkage disequilibrium with an unrecognized (not genotyped on the IBC array) causal variant in African Americans; this causal variant may not be present in European Americans, or it may be present but in poor linkage disequilibrium with rs2515629 in European Americans due to differences in correlation structure.

Third, in some gene regions there are differences in the distributions of independent lipid-associated SNPs. Presumably these independent SNPs reflect the presence of independent causal variants. For example, in the *CETP* locus we identified at least 3 independently associated SNPs (with HDL) in African Americans and 4 independently associated SNPs in European Americans (Table 2), scattered over a range of 20 kb, with none clearly marking the same causal variants. Similar complexity is seen in the *PCSK9*, *LPL*, and *APOE* loci and, to a lesser degree, in most of the other 11 loci for which we performed conditional analyses (Figures S1 and S2). Thus, significant variability in the full spectrum of causal DNA variants across gene regions in different ethnic groups may well be the rule rather than the exception.

A previous analysis of lipid-associated loci in the Jackson Heart Study identified several loci–*LPL*, *APOB*, and *GCKR*–that appeared to account for some part of the inter-ethnic variation in lipid profiles, and found that SNPs in the *LPL* locus displayed different effect sizes depending on whether they existed on a background of European vs. African ancestry [18]. Our study found evidence of a similar phenomenon for a larger number of loci. In the *LPL* locus, for example, the most highly associated SNPs for TG in either European Americans and African Americans both have larger estimated effect sizes in the latter than the former (rs3916027, β = –0.089 vs. β = –0.0072; rs327, β = 0.097 vs. β = 0.0026), consistent with what was observed in the previous study. Other loci in which specific variants appear to have larger effect sizes in African Americans than in European Americans include *PCSK9* for LDL-C (rs11806638, β = –0.11 vs. β = –0.054), *GCKR* for TG (rs1260326, β = 0.088 vs. β = 0.037), *APOA1-C3-A4-A5* for TG (rs2075290, β = –0.12 vs. β = –0.064; rs9804646, β = –0.083 vs. β = –0.039), and *LCAT* for HDL-C (β = 0.11 vs. β = 0.069), among others. Similarly, there are a number of loci in which specific variants appear to have larger effect sizes in European Americans than in African Americans (Table 1). Thus, genetic differences in lipid determinants between the two ethnicities appear to be widespread across many loci.

### Inter-ethnic Comparisons of SNP-phenotype Relationships may Pinpoint Causal DNA Variants

Finally, while it has been assumed that differing patterns of linkage disequilibrium in European Americans and African Americans could be helpful in localizing shared, causal DNA variants, for the reasons stated above this may not often be the case. Of the 29 loci we studied, only in 4 cases did association signals in the two ethnicities appear to unambiguously converge (Figures S1 and S2).

In the *SORT1* locus, the most highly associated SNPs in the two ethnicities were not identical but were in close proximity (within a few kb) and in high linkage disequilibrium, suggesting that they are marking the same causal variant (Figure S1A). In European Americans, we found six SNPs that together bear the strongest association with LDL-C (*P* values ranging from 1.69×10^−51^ to 1.33×10^−49^) and are effectively in perfect linkage disequilibrium. In contrast, for the same 6 SNPs in African Americans, they are in varying degrees of linkage disequilibrium, with rs12740374 standing out as having the strongest association (*P* = 9.33×10^−20^ in African Americans; *P* = 2.90×10^−51^ in European Americans). Thus, this analysis nominates rs12740374 as a strong candidate for the causal variant in the locus. Of note, a recent study identified rs12740374 as a causal variant through functional experimentation in cell-based reporter assays [19], confirming the potential value of inter-ethnic comparisons of SNP-phenotype relationships in identifying causal DNA variants.

In the *LDLR* and *LIPC* loci, respectively, rs6511720 and rs2070895 were the most highly associated SNPs in both ethnicities, suggesting that each is either a causal SNP or tightly linked to a causal SNP (Figure S1B, S1C). In the *APOB* locus, the most highly LDL-C-associated SNP in European Americans, rs934197, is not associated with LDL-C in African Americans despite being common in the second ethnic group (Figure S1D). However, the second independent SNP in the locus found in European Americans, rs562338, is the most highly LDL-C-associated SNP in African Americans, suggesting that it may be a causal variant in the *APOB* locus, albeit not the only one detected in European Americans.

Rs6511720 is located in intron 1 of the *LDLR* gene, suggesting that it might affect either transcription or splicing of the gene transcript. Rs2070895 is in the promoter region of *LIPC*, just 300 bp upstream of the start codon, suggesting a role in gene transcription. Finally, rs562338 is located 20 kb upstream of the *APOB* gene, which would suggest some long-range regulatory role. Further experimentation aimed at evaluating whether rs6511720, rs2070895, and rs562338 are indeed causal DNA variants and by what mechanisms they might act in their respective loci is warranted.

## Supporting Information

Figure S1 **Graphical plots of SNPs in four lipid-associated loci in which the best independently associated SNPs for European Americans and African Americans coincide.** The x-axis indicates the basepair position on the indicated chromosome. The y-axis indicates the negative log of the *P* value of association with the indicated lipid trait. Red stars indicate the *P* values of association of the SNPs in European Americans, black circles in African Americans. Each of the best independently associated SNPs in each locus is labeled, and all of the SNPs in strong linkage disequilibrium with one of the best SNPs are enclosed in the same oval.
(DOC)

Figure S2 **Graphical plots of SNPs in each lipid-associated locus.** The x-axis indicates the basepair position on the indicated chromosome. The y-axis indicates the negative log of the *P* value of association with the indicated lipid trait. Red stars indicate the *P* values of association of the SNPs in European Americans, black circles in African Americans. Each of the best independently associated SNPs in each locus is circled and labeled.
(DOC)

Table S1 **CARe participant characteristics.**
(DOC)

Table S2 **Hardy-Weinberg** ***P*** **values for lipid-associated SNP variants.**
(DOC)

Table S3 **SNP×SNP interactions between the most significant SNPs at each LDL-C-related locus among European Americans.**
(DOC)

Table S4 **SNP×SNP interactions between the most significant SNPs at each LDL-C-related locus among African Americans.**
(DOC)

Table S5 **SNP×SNP interactions between the most significant SNPs at each HDL-C-related locus among European Americans.**
(DOC)

Table S6 **SNP×SNP interactions between the most significant SNPs at each HDL-C-related locus among African Americans.**
(DOC)

Table S7 **SNP×SNP interactions between the most significant SNPs at each triglyceride-related locus among European Americans.**
(DOC)

Table S8 **SNP×SNP interactions between the most significant SNPs at each triglyceride-related locus among African Americans.**
(DOC)

Table S9 **SNP×sex interaction tests for the most significant SNPs at each LDL-C-related locus.**
(DOC)

Table S10 **SNP×sex interaction tests for the most significant SNPs at each HDL-C-related locus.**
(DOC)

Table S11 **SNP×sex interaction tests for the most significant SNPs at each triglyceride-related locus.**
(DOC)

Table S12 **Top ten significant SNP×sex interactions for each lipid trait among European Americans.**
(DOC)

Table S13 **Top ten significant SNP×sex interactions for each lipid trait among African Americans.**
(DOC)
