# Supplementary figures and images for: Multi-Ethnic Analysis of Lipid-Associated Loci: The NHLBI CARe Project
Source: PLoS One. 2012 May 21;7(5):e36473. doi: 10.1371/journal.pone.0036473 (PMC3357427; doi:10.1371/journal.pone.0036473)

Figure S1.


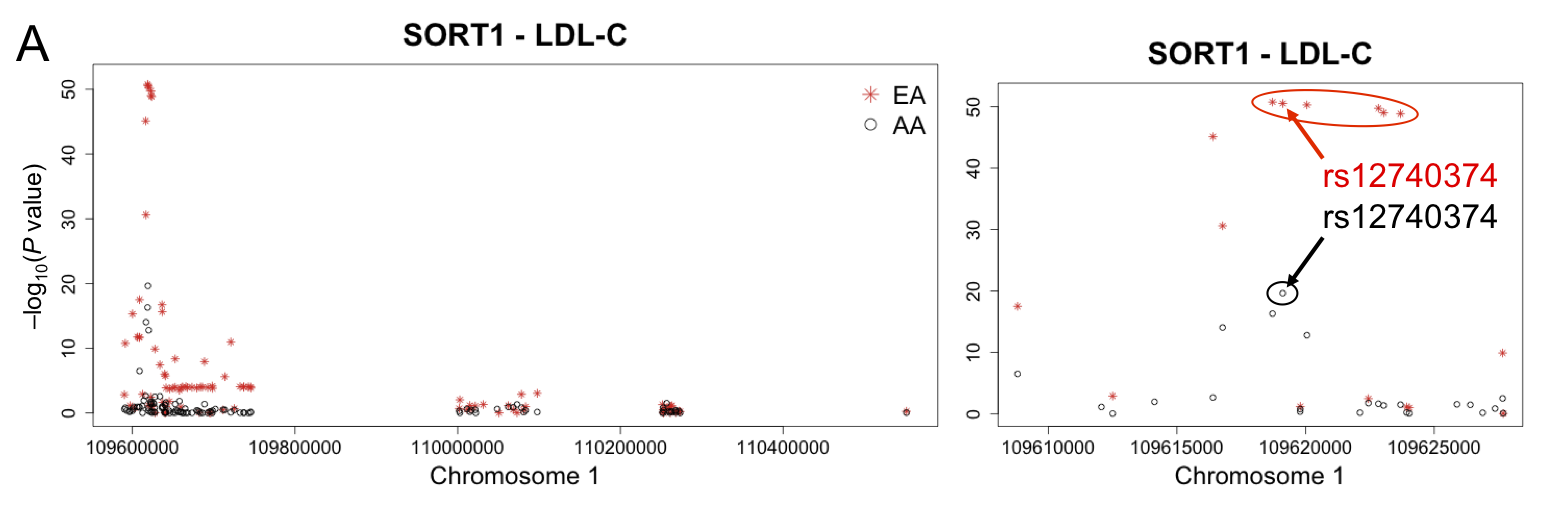

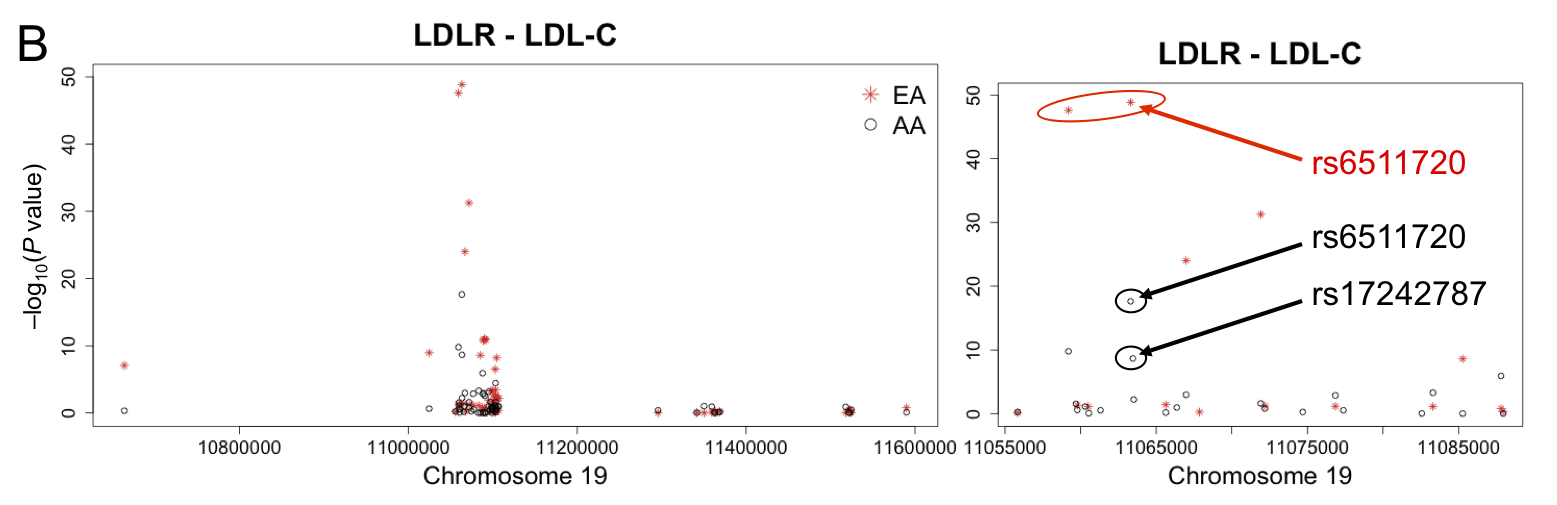

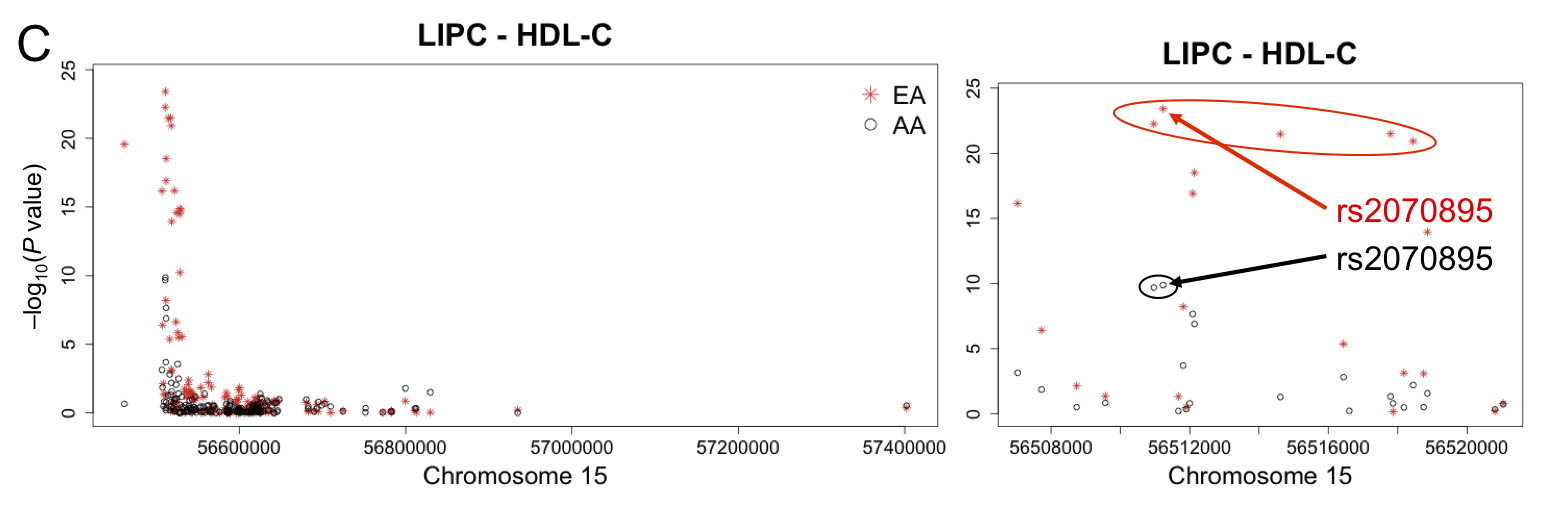

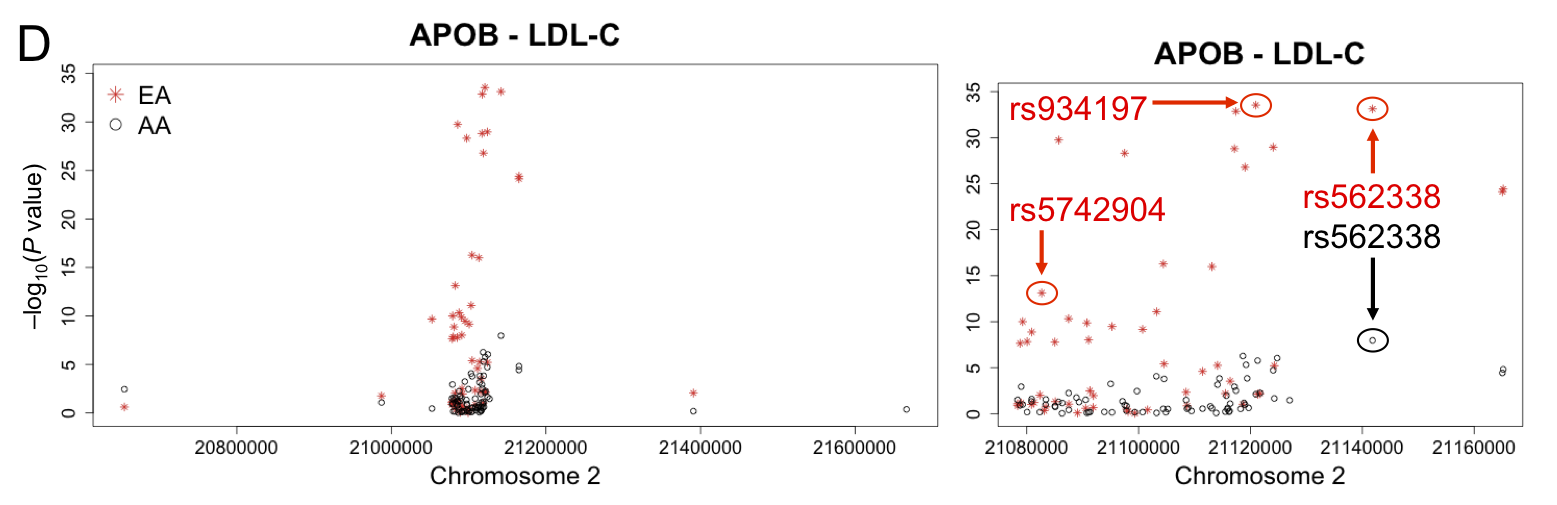

Supplement: Figure S1 — Graphical plots of SNPs in four lipid-associated loci in which the best independently associated SNPs for European Americans and African Americans coincide. The x-axis indicates the basepair position on the indicated chromosome. The y-axis indicates the negative log of the P value of association with the indicated lipid trait. Red stars indicate the P values of association of the SNPs in European Americans, black circles in African Americans. Each of the best independently associated SNPs in each locus is labeled, and all of the SNPs in strong linkage disequilibrium with one of the best SNPs are enclosed in the same oval. (DOC) [file pone.0036473.s001.doc]

Figure S2.


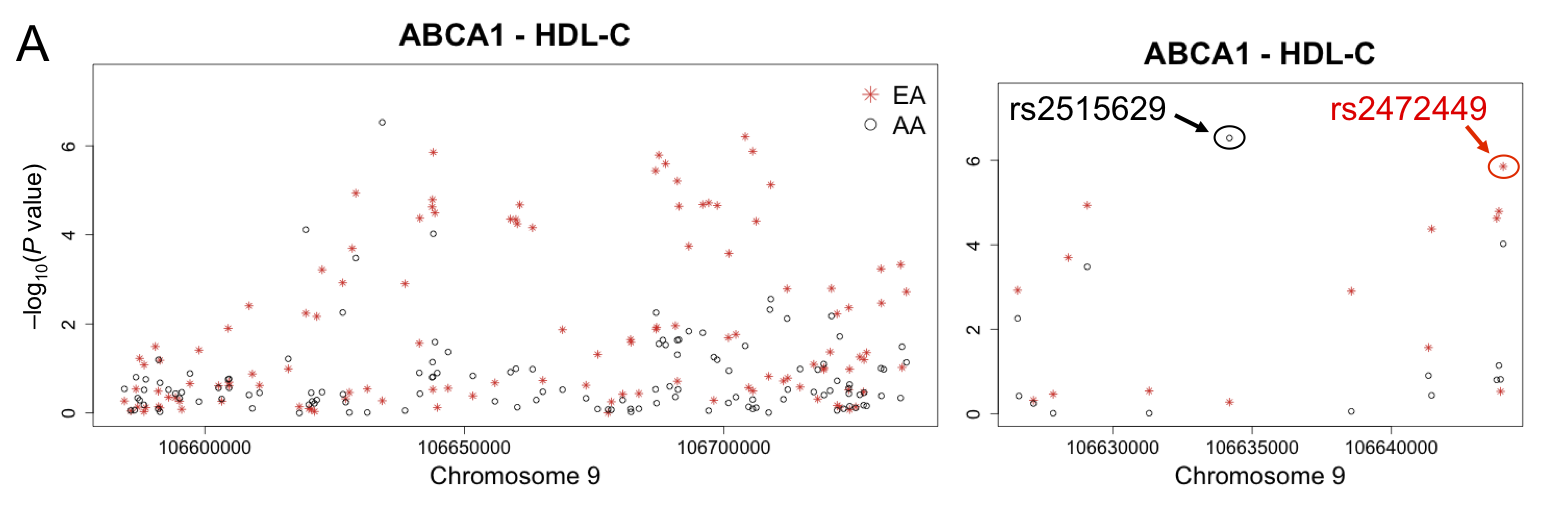


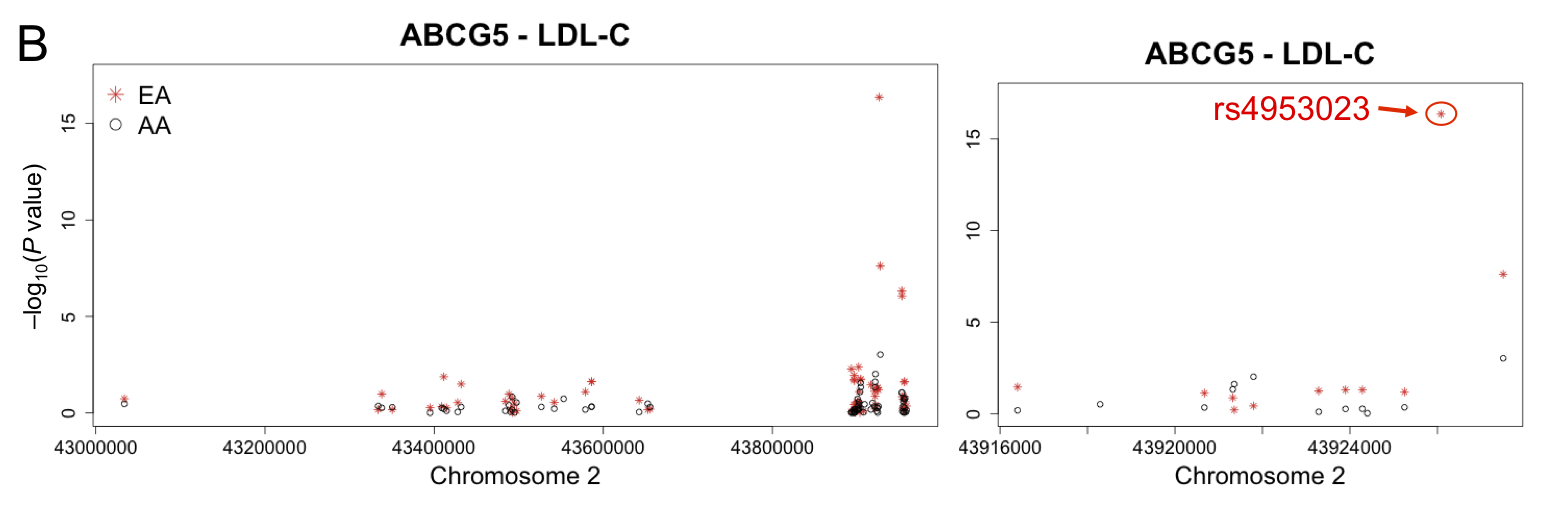


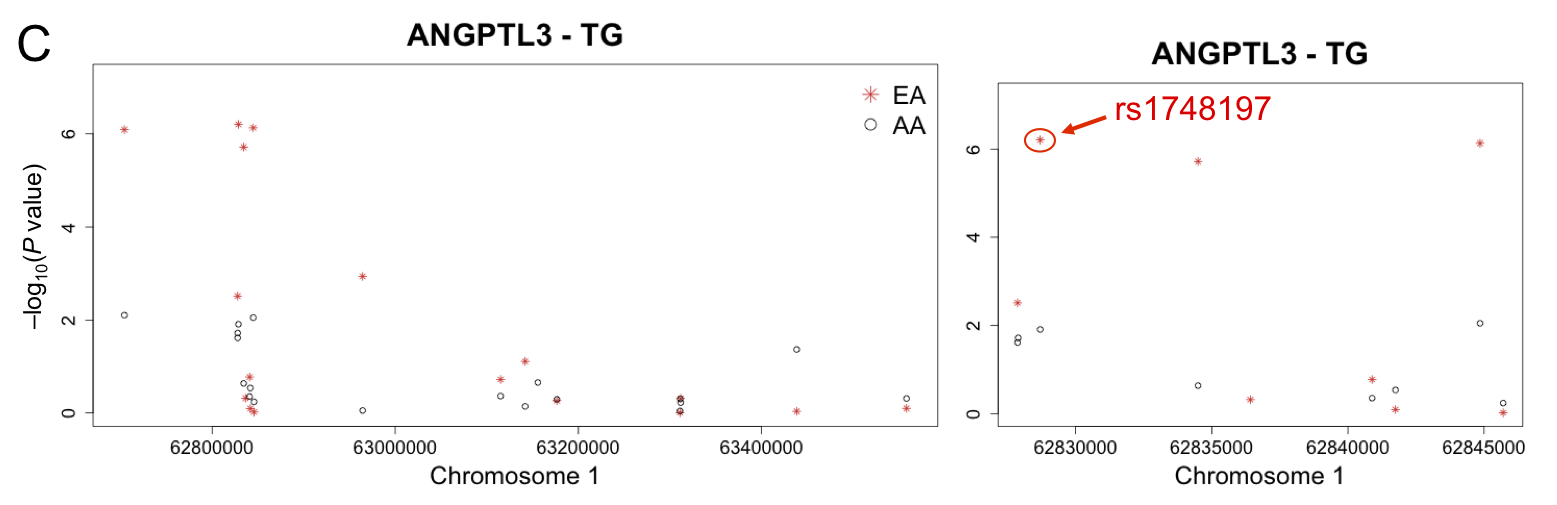


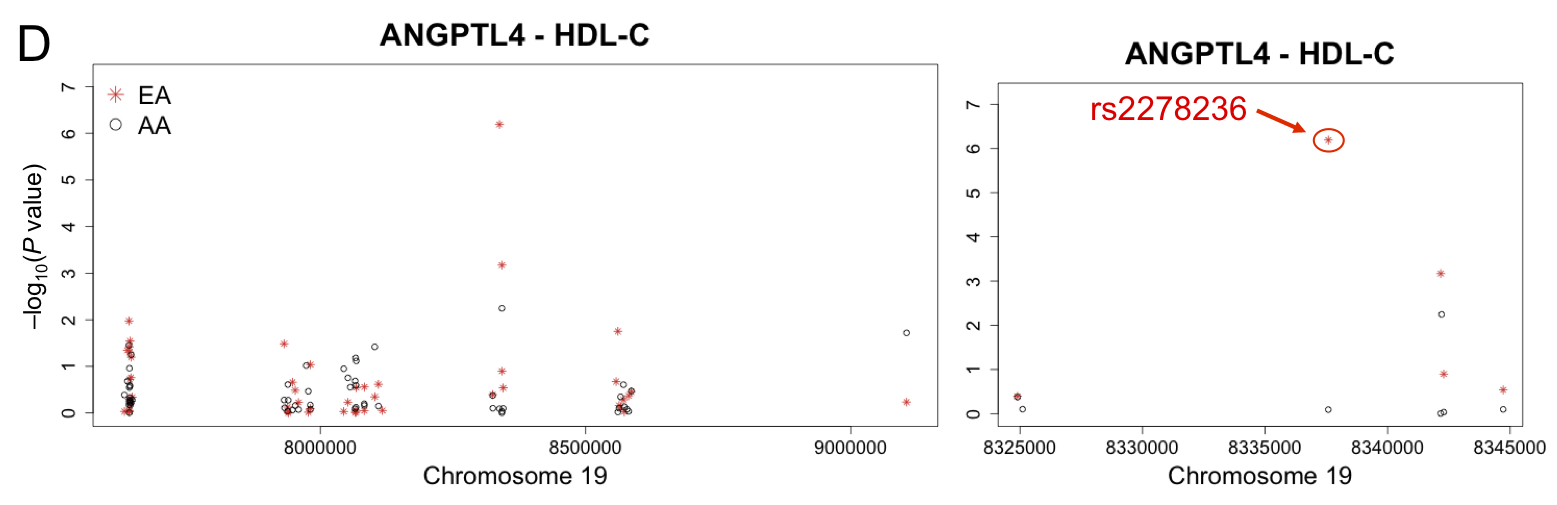


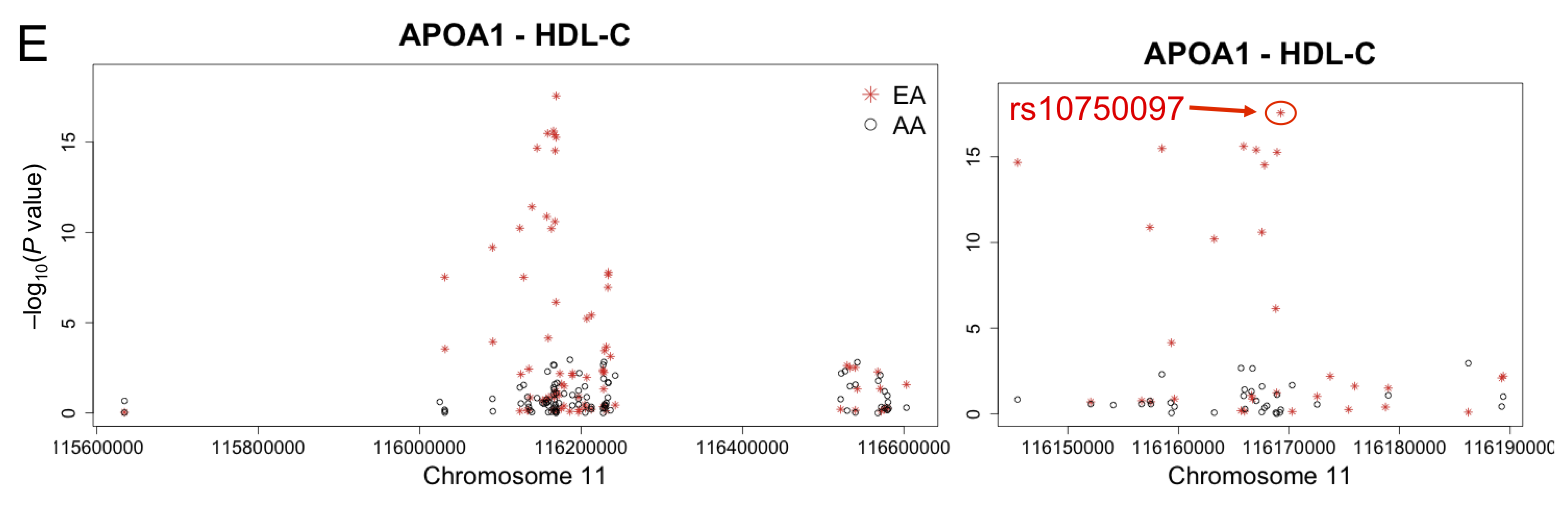

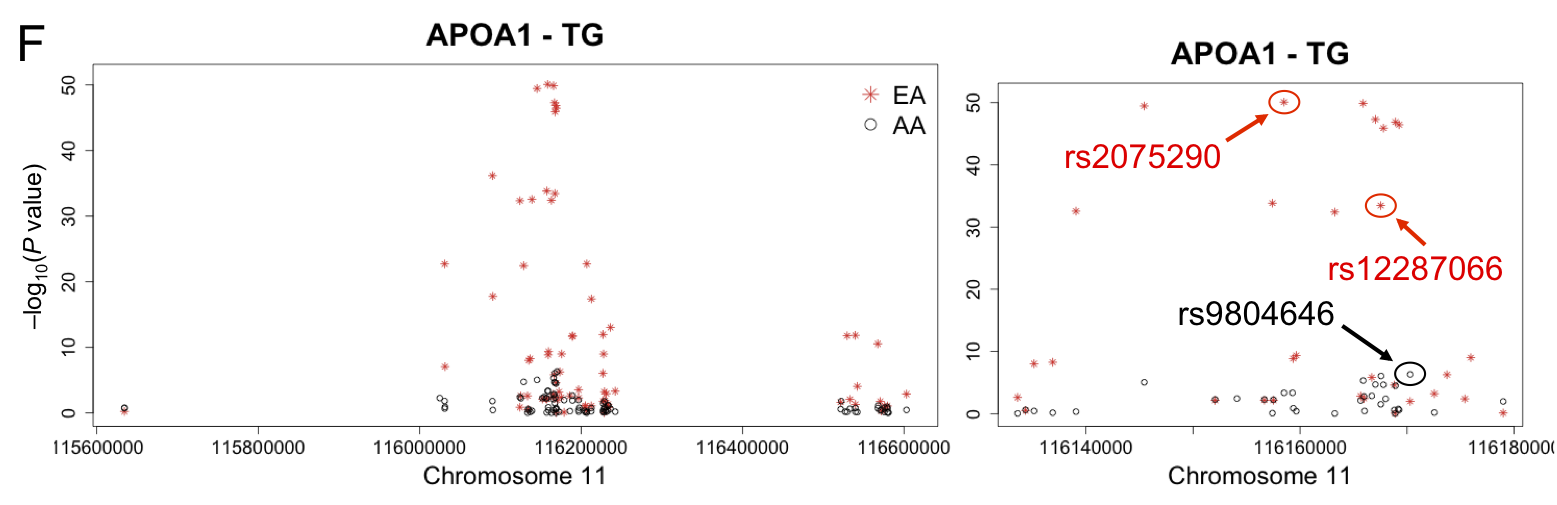

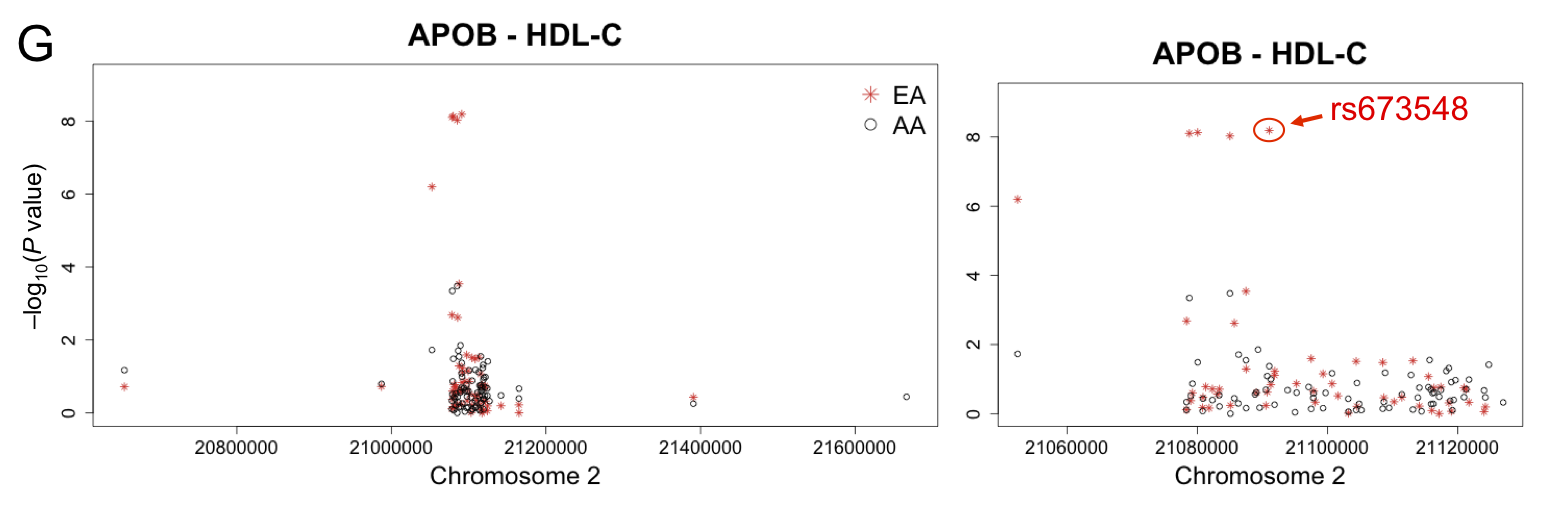

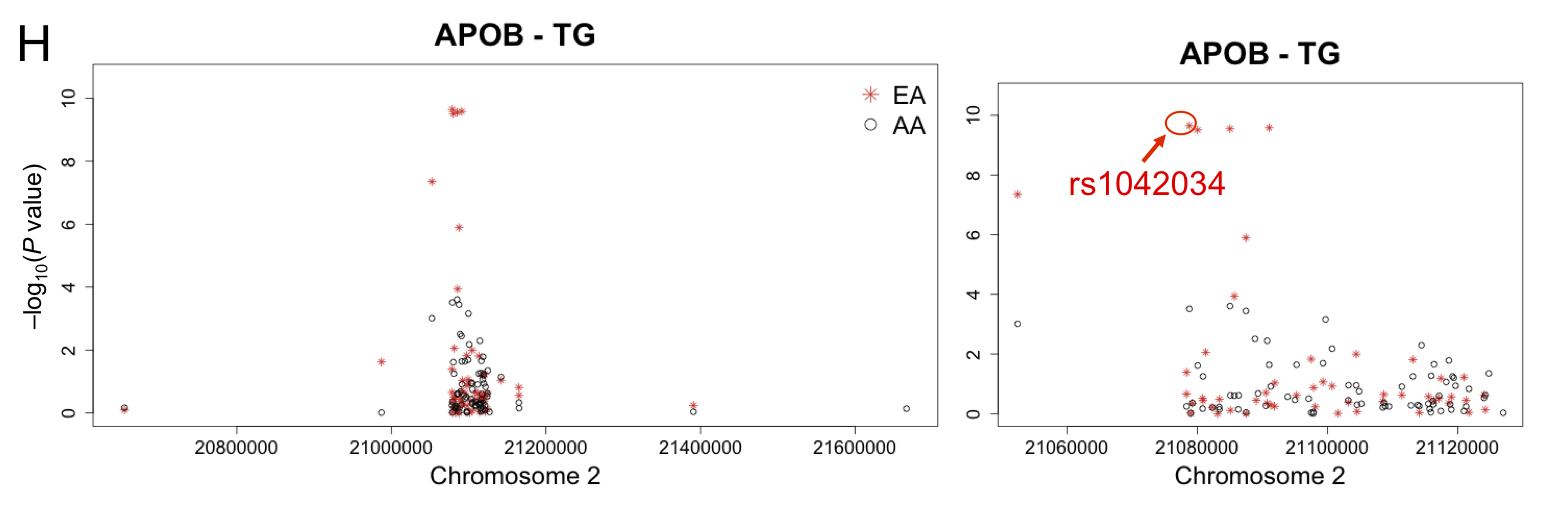

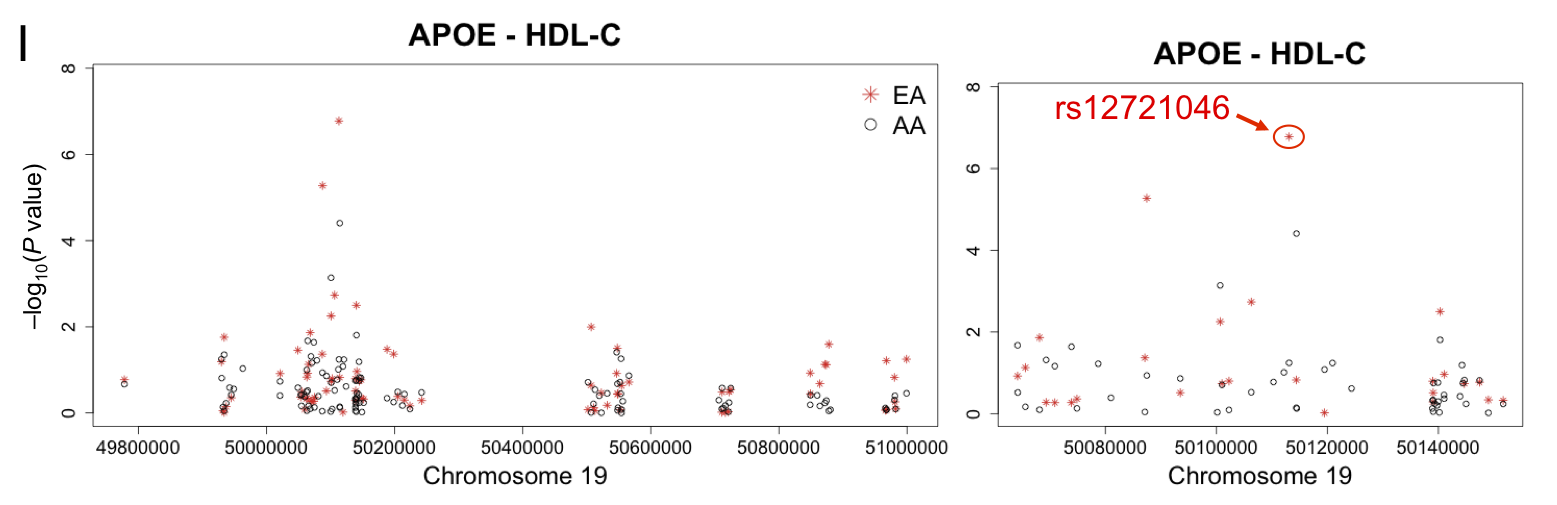

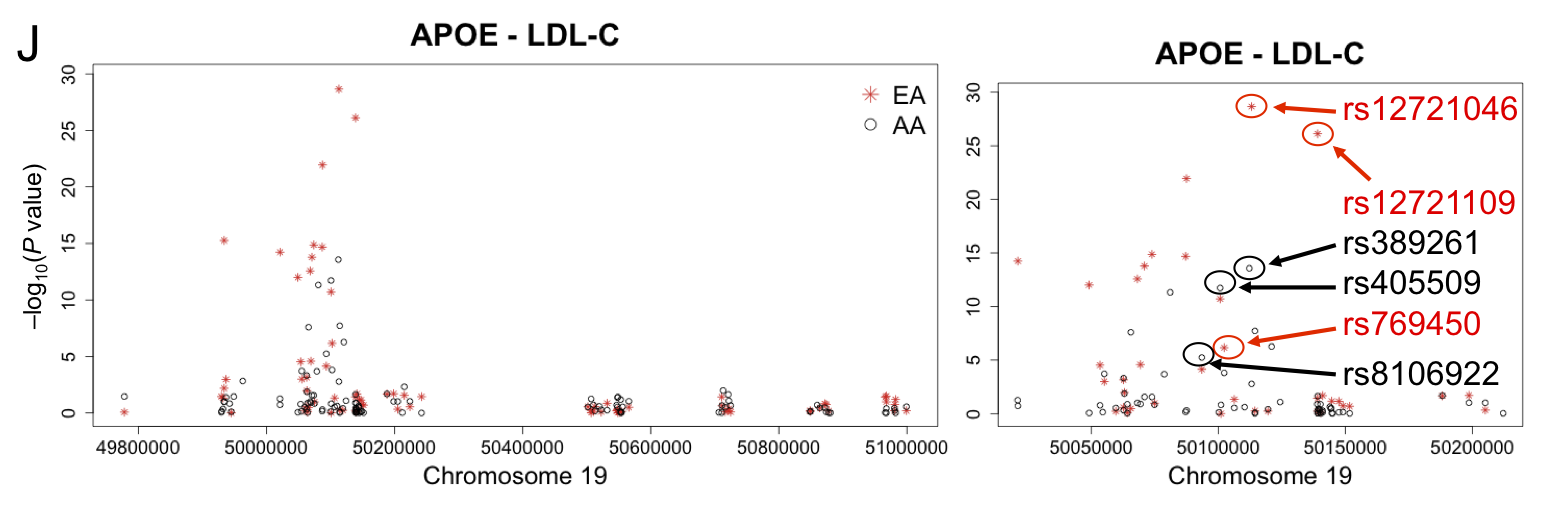

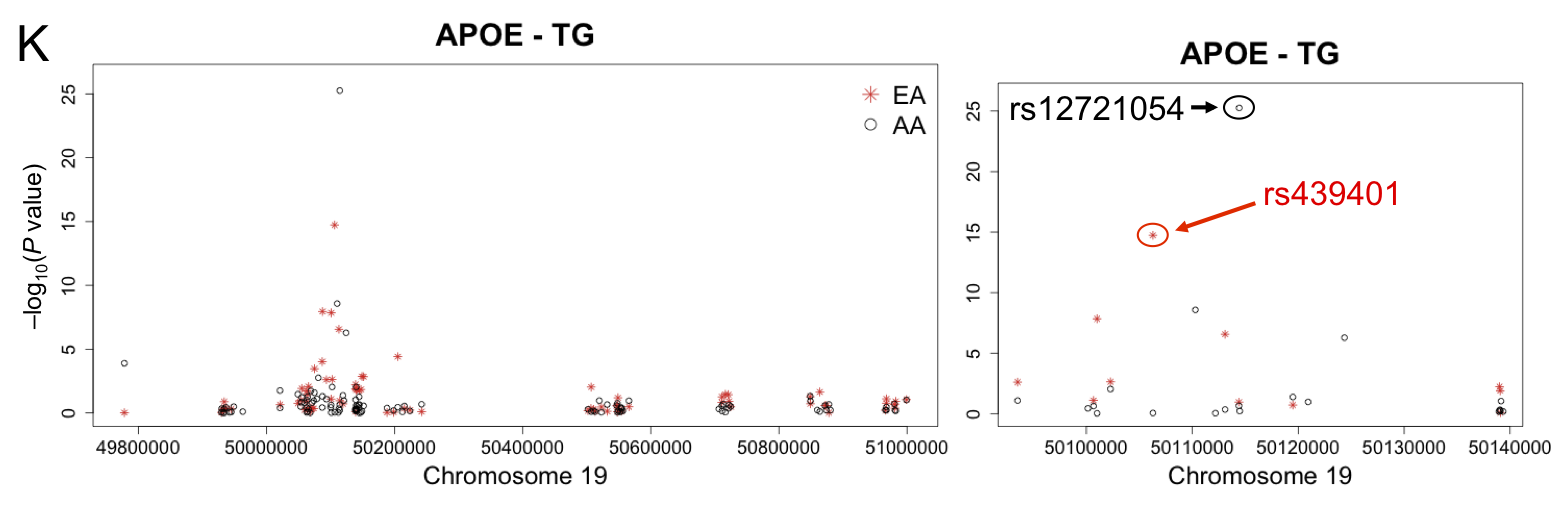

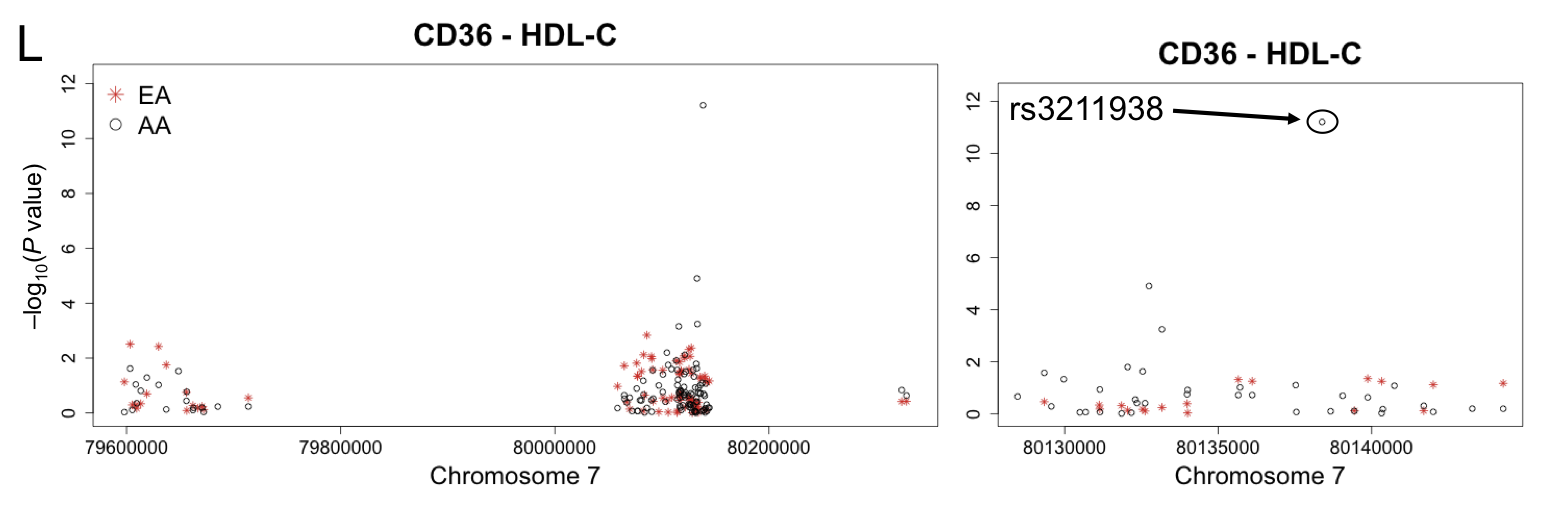

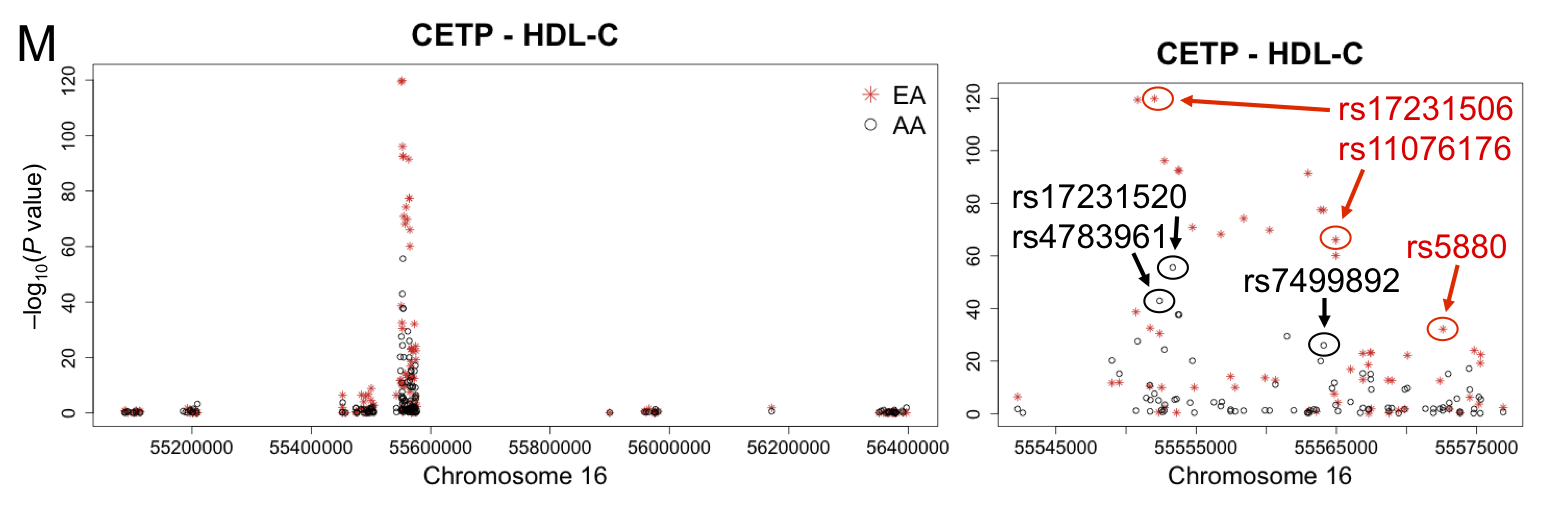

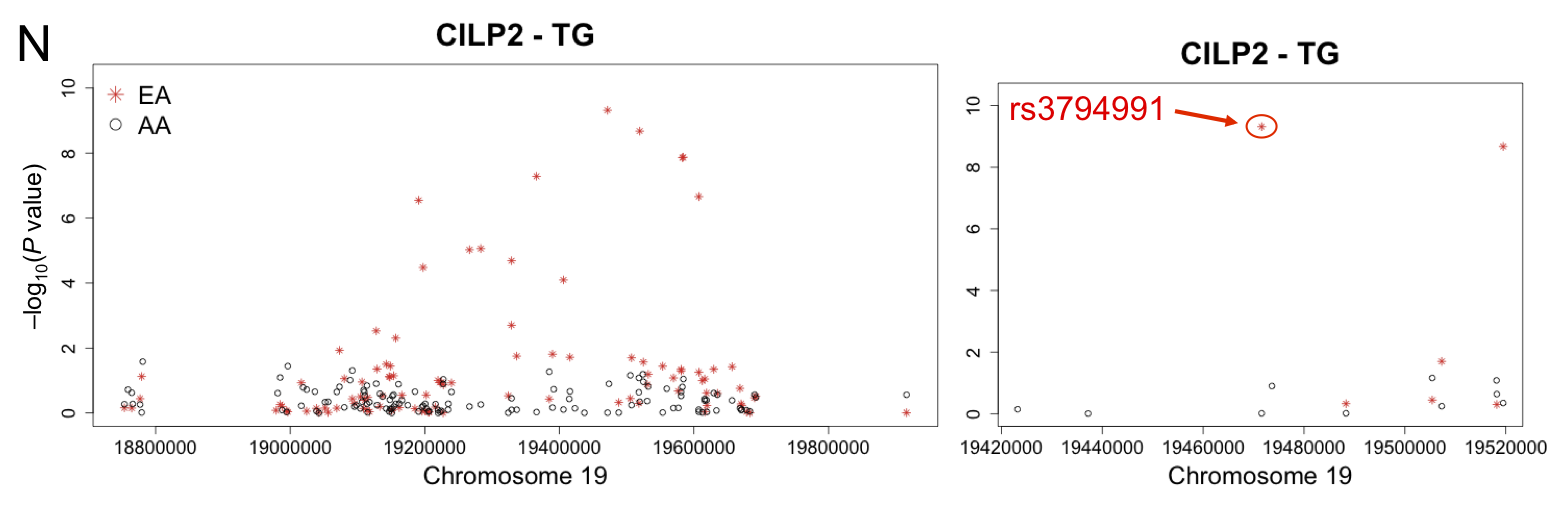

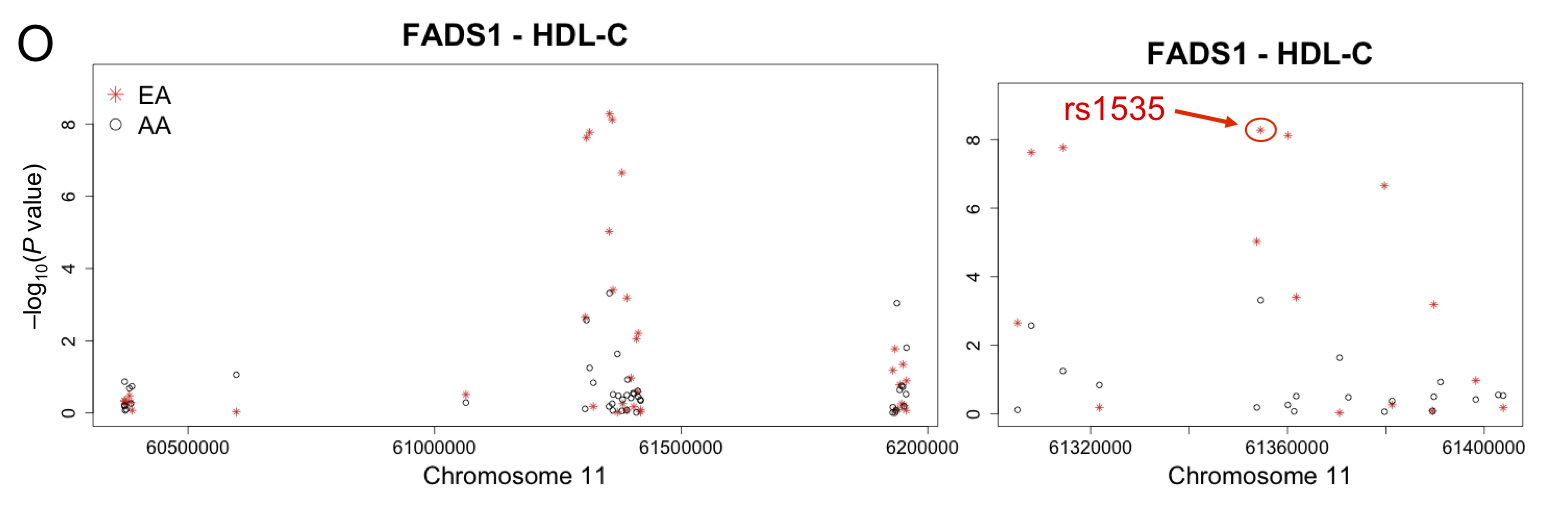

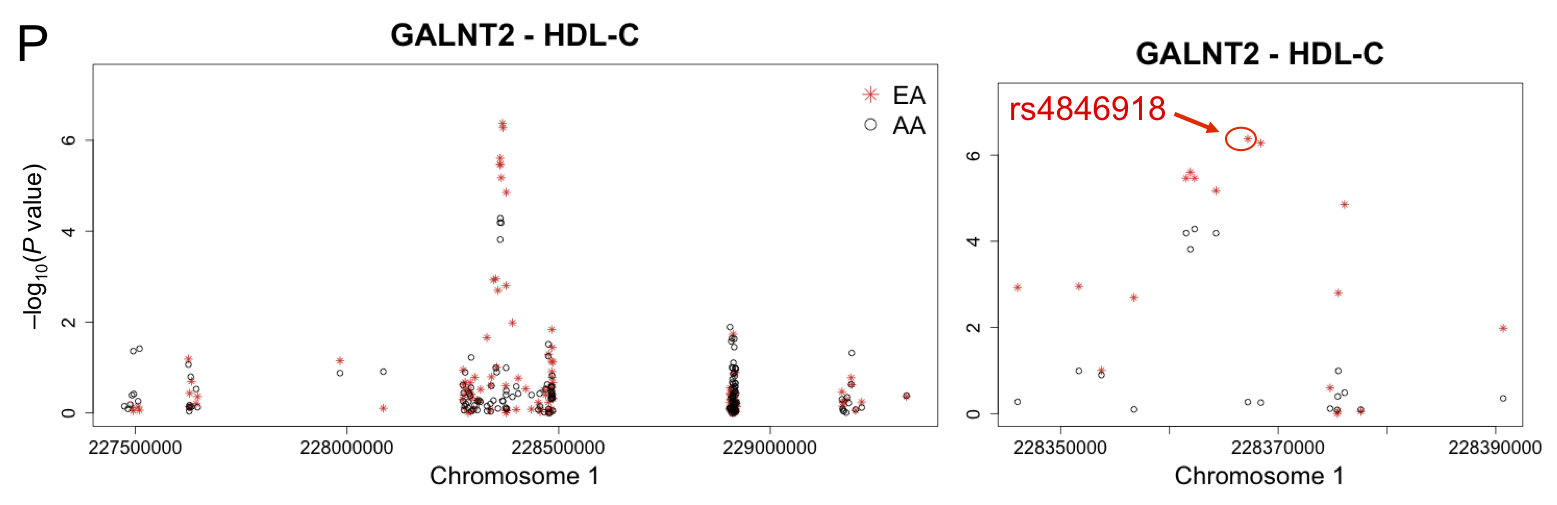

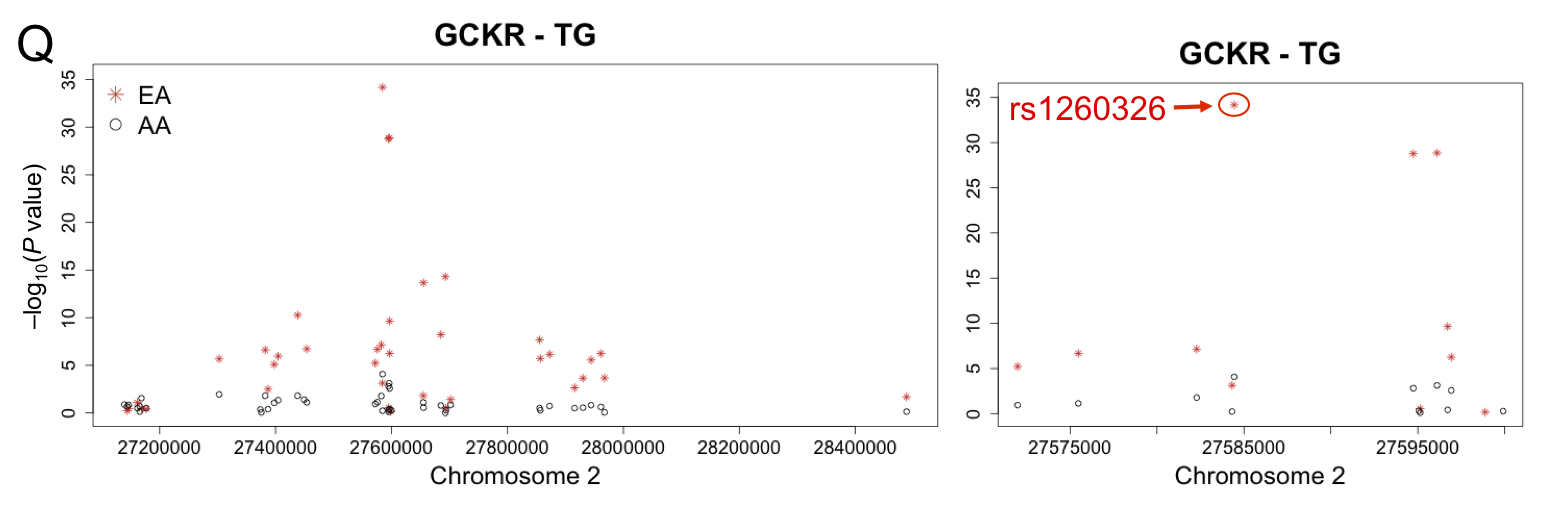

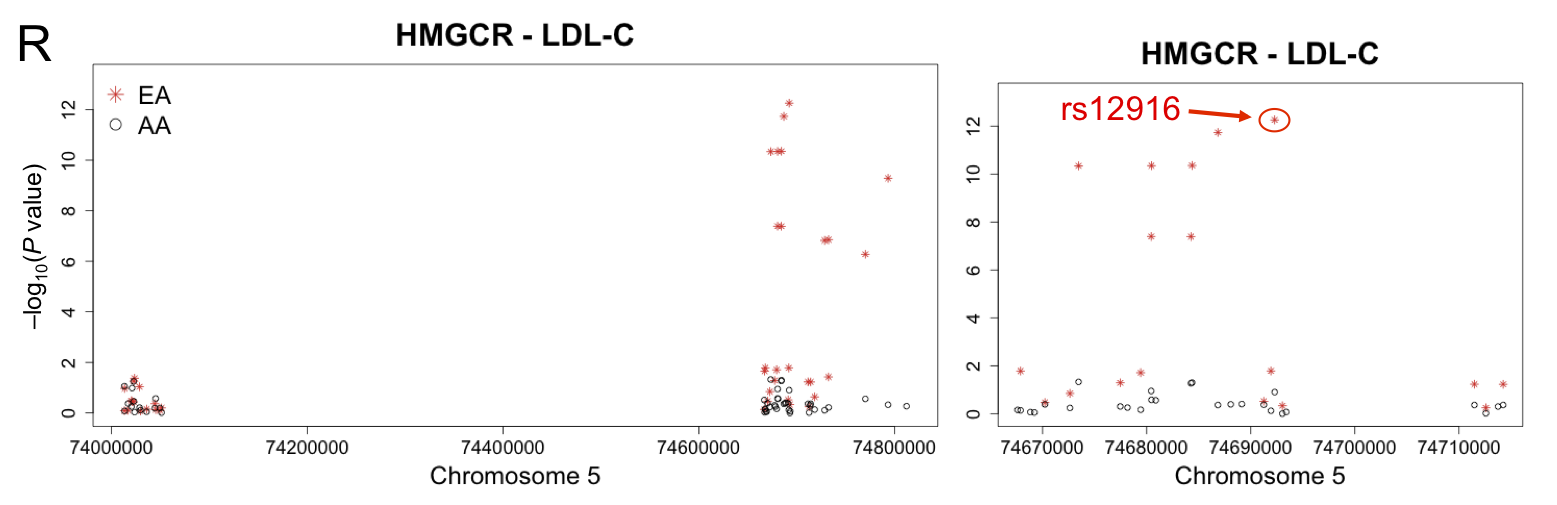

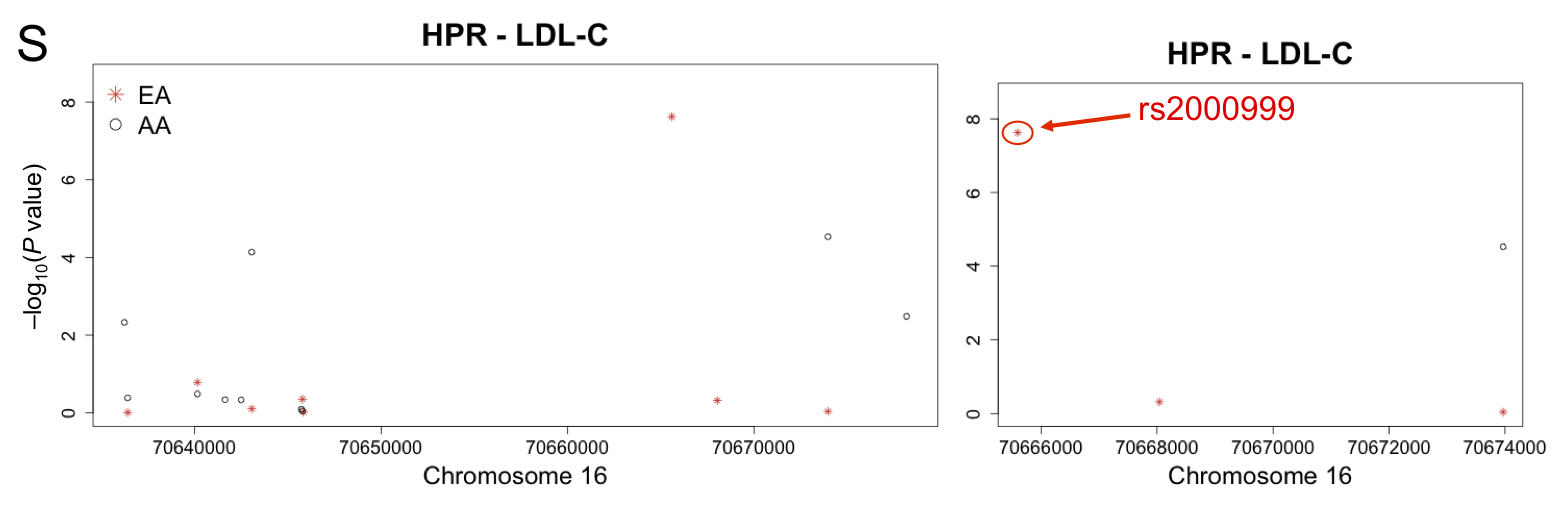

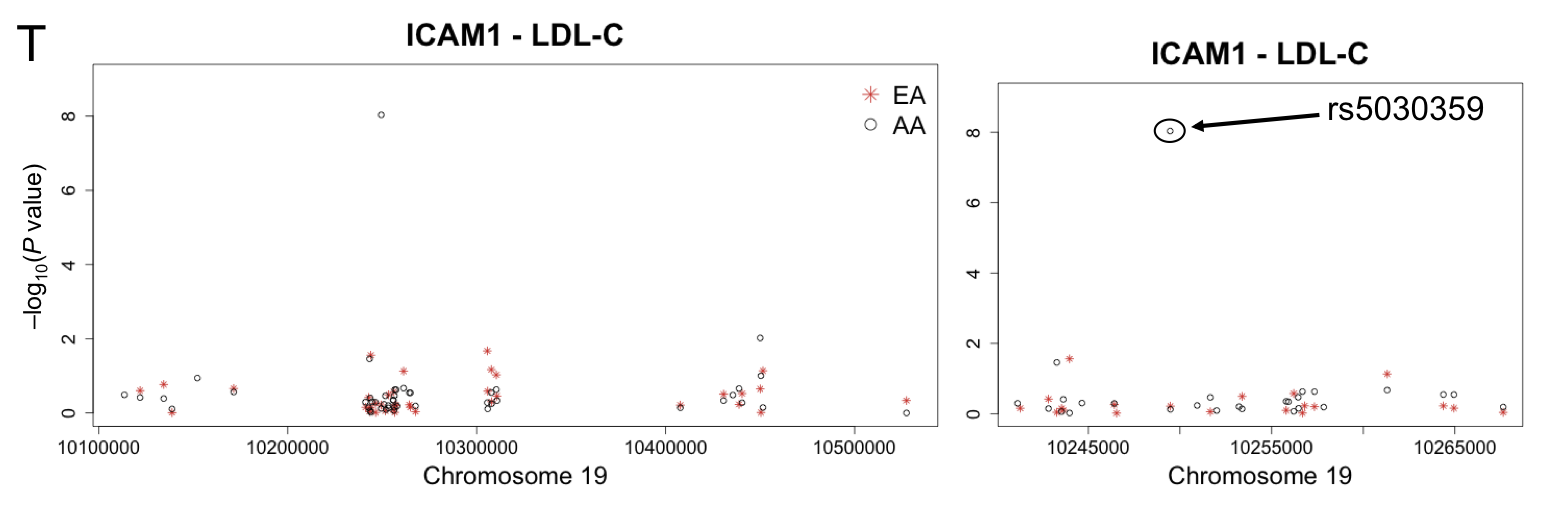

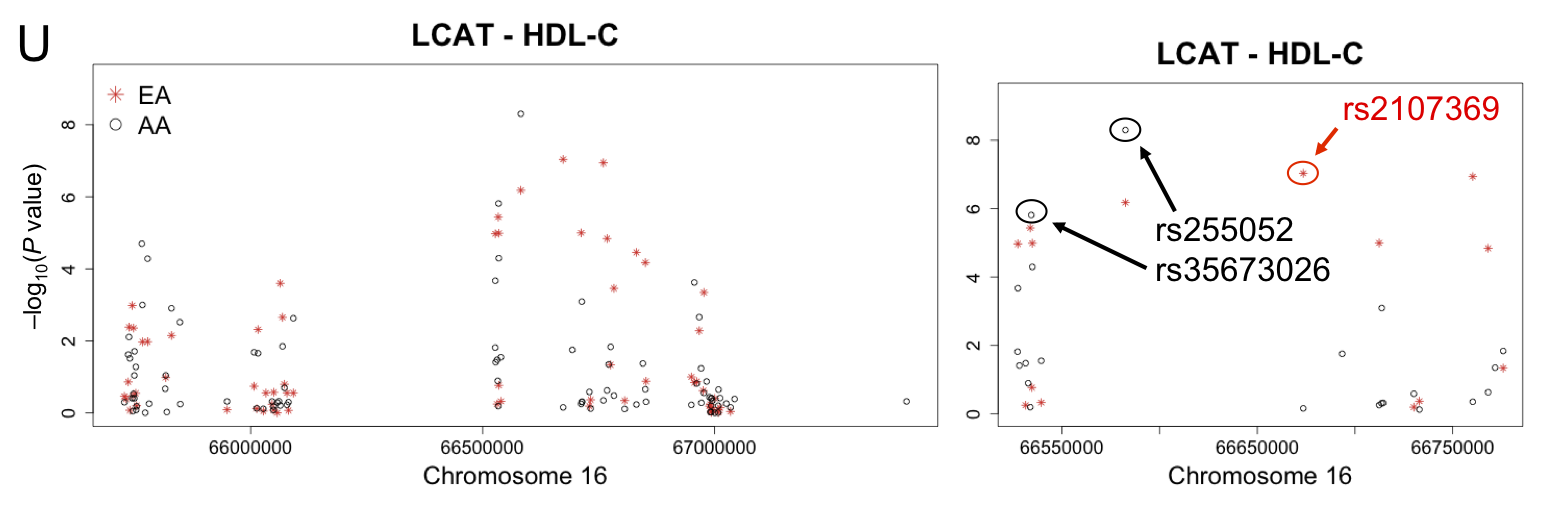

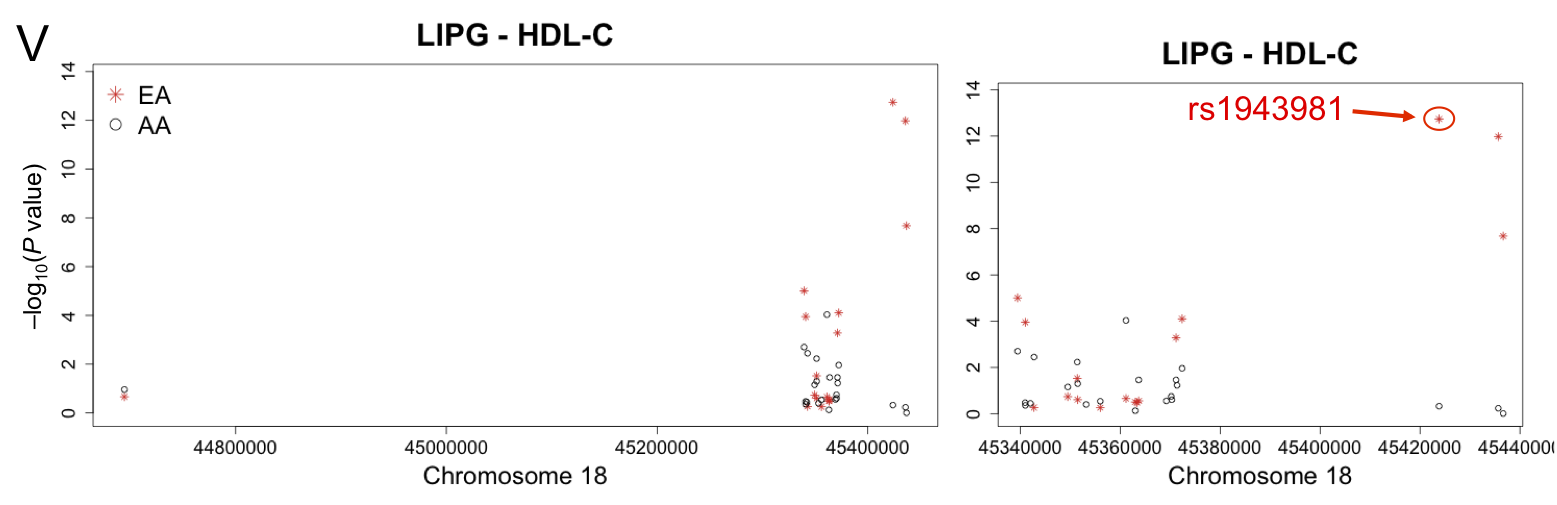

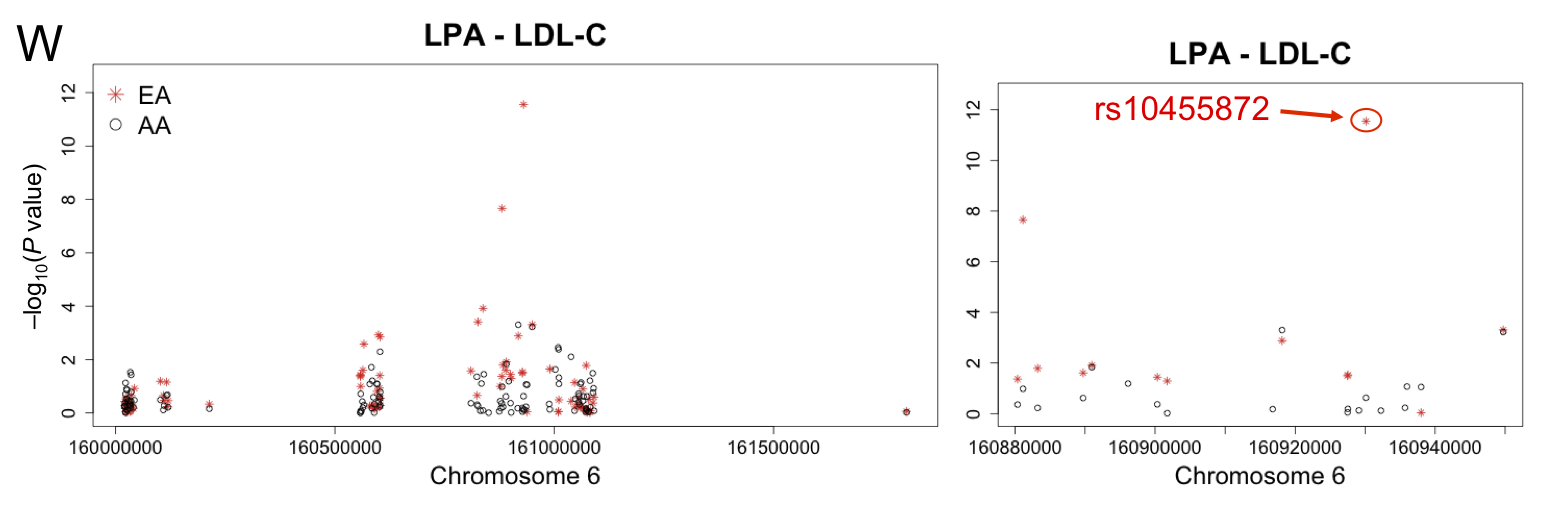

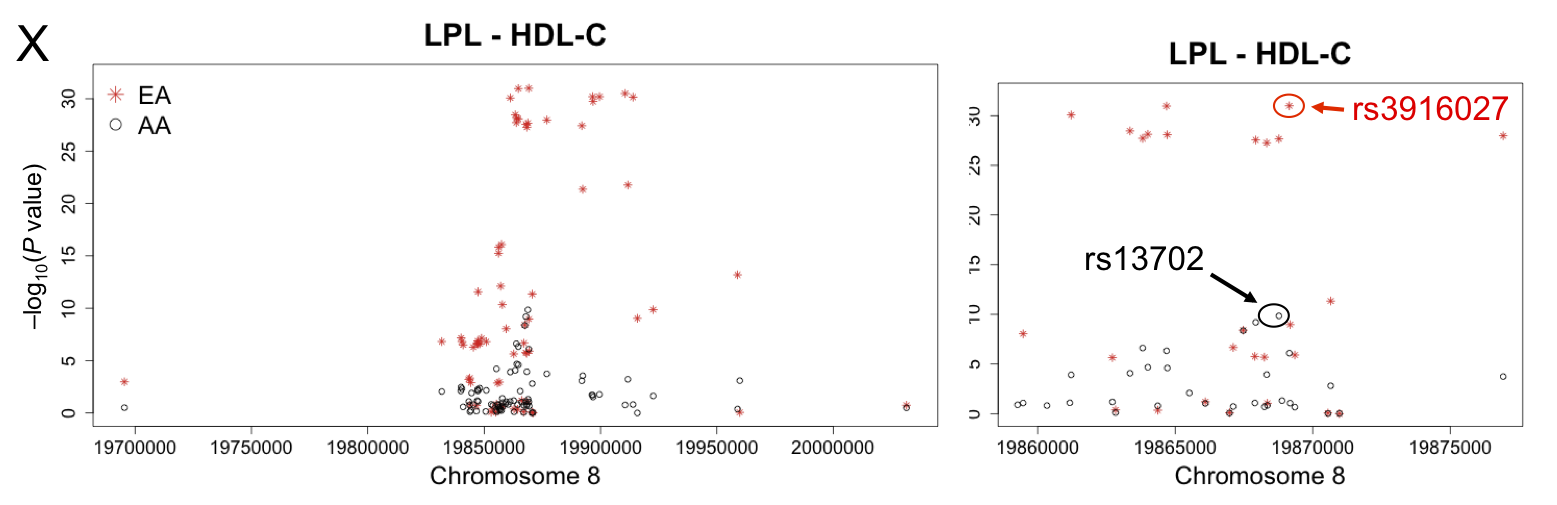

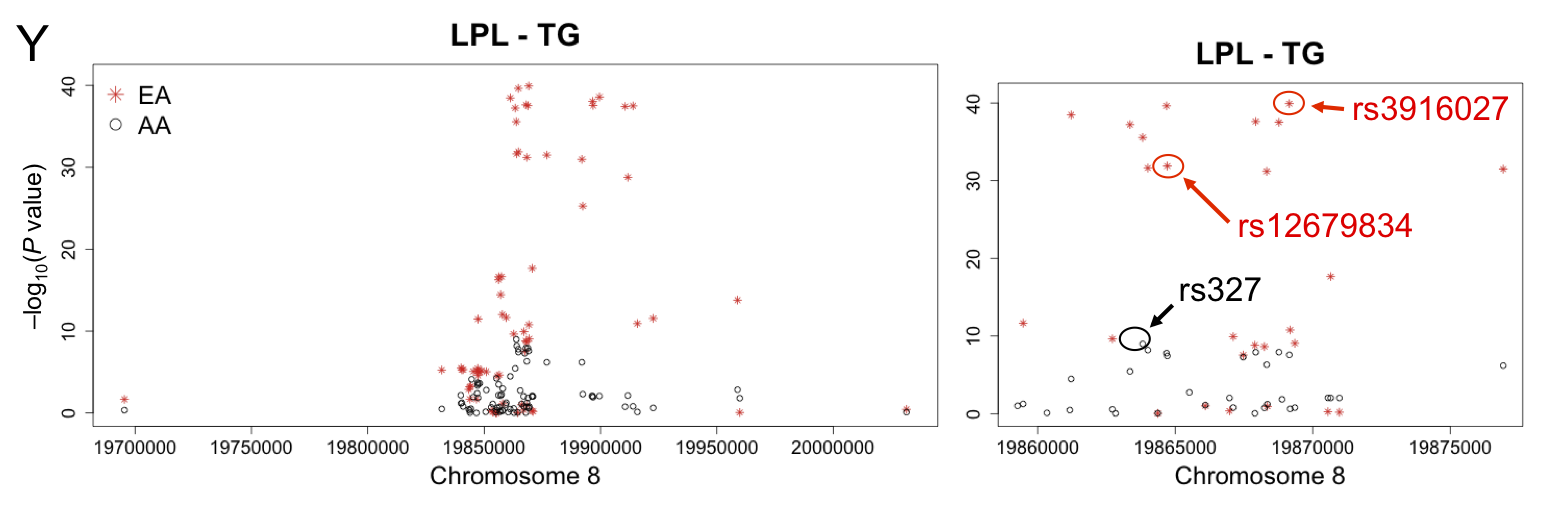

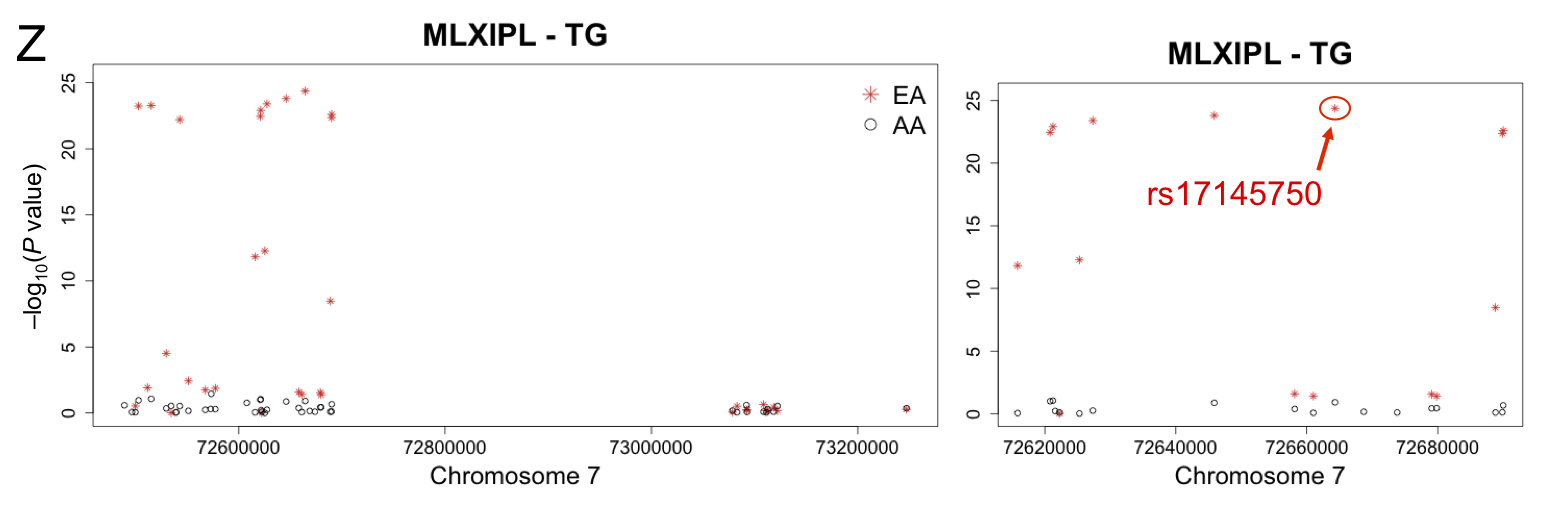

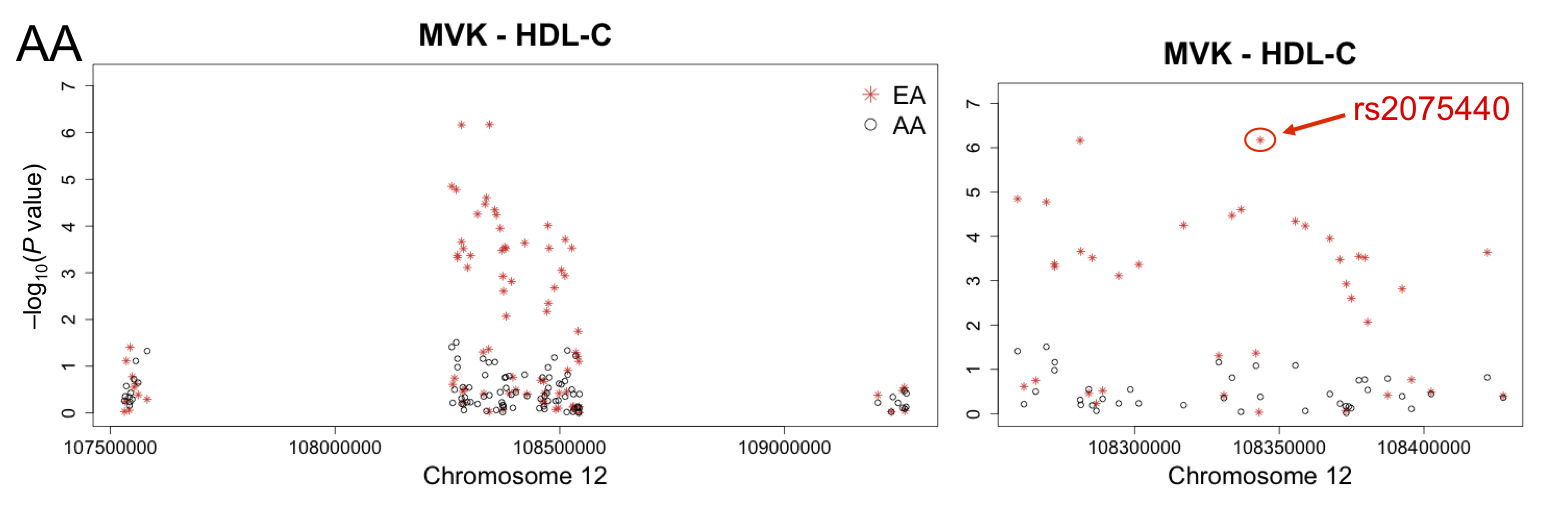

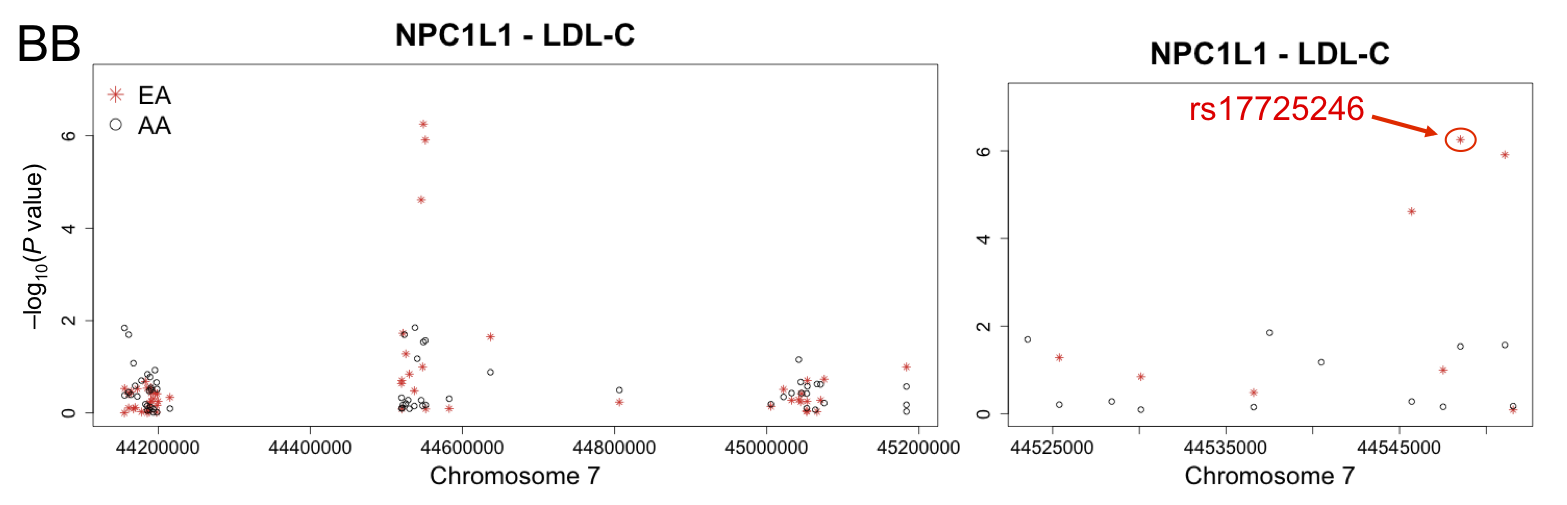

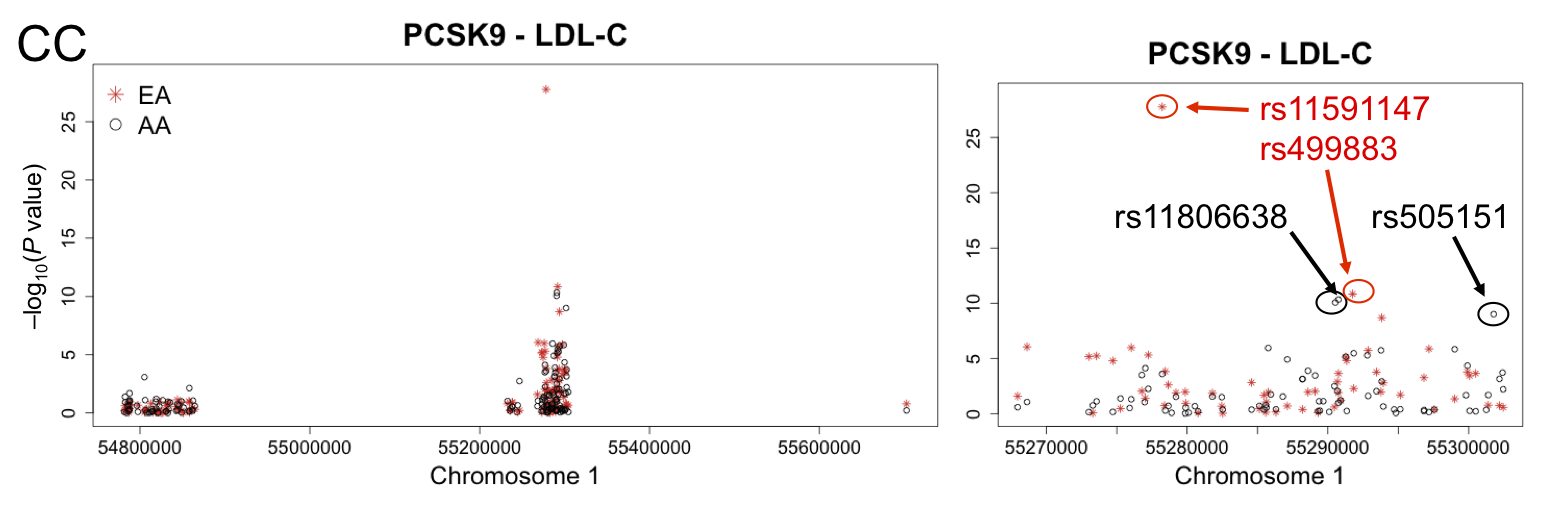

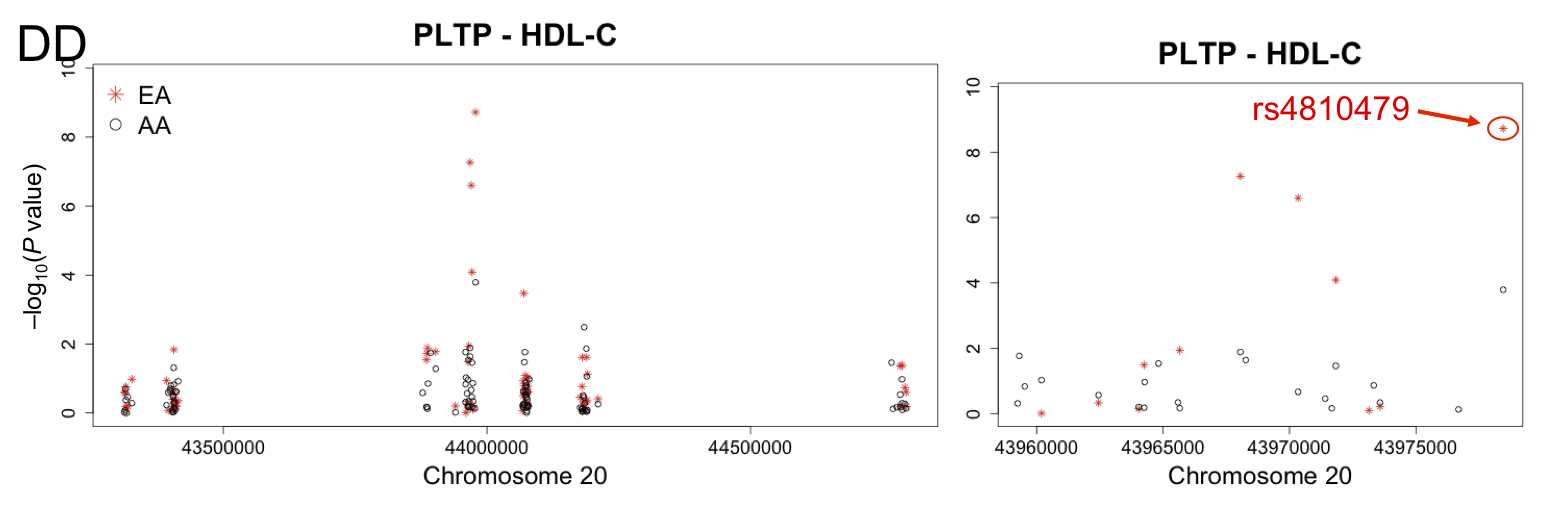

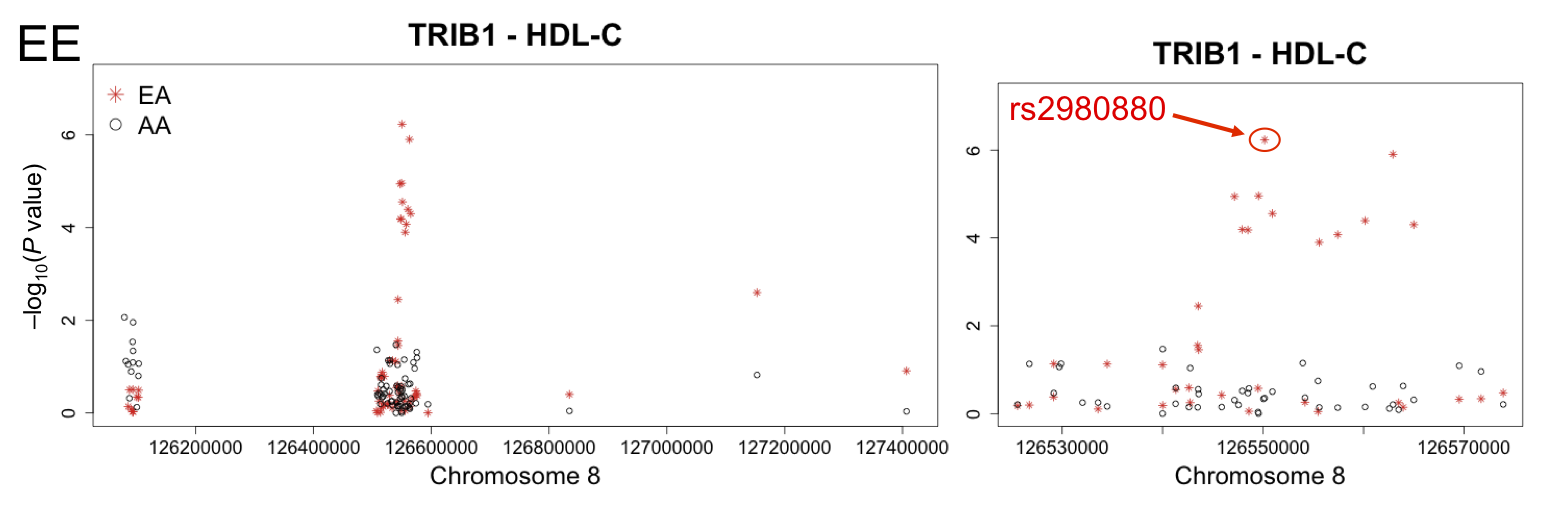

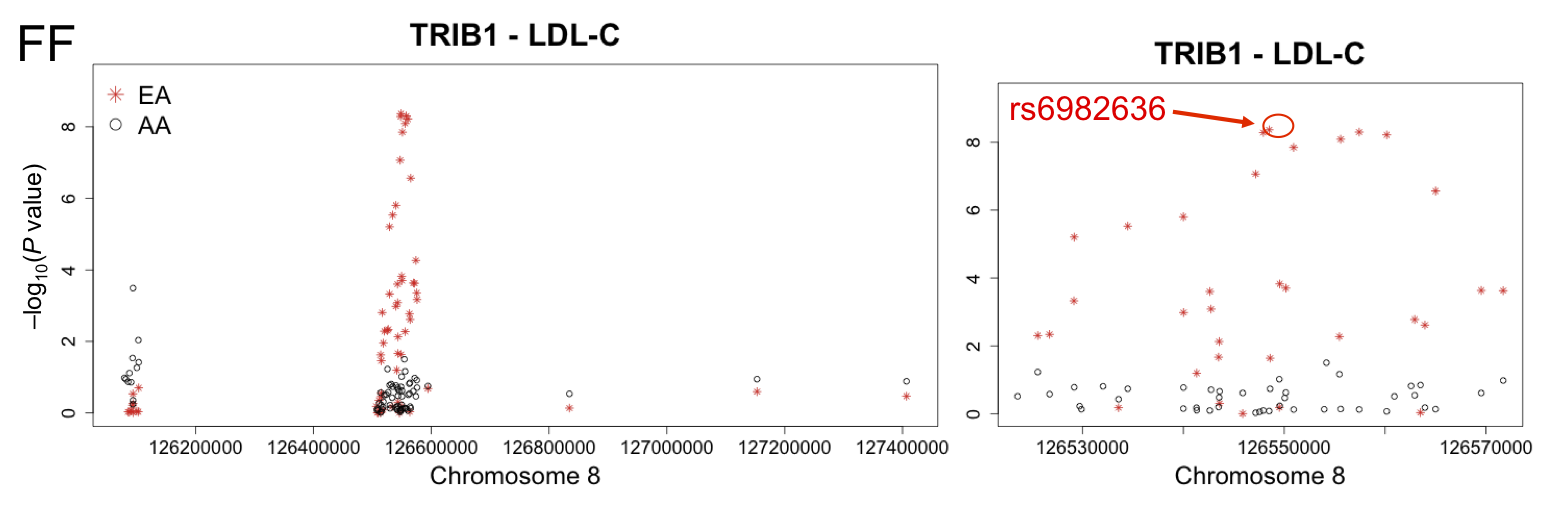

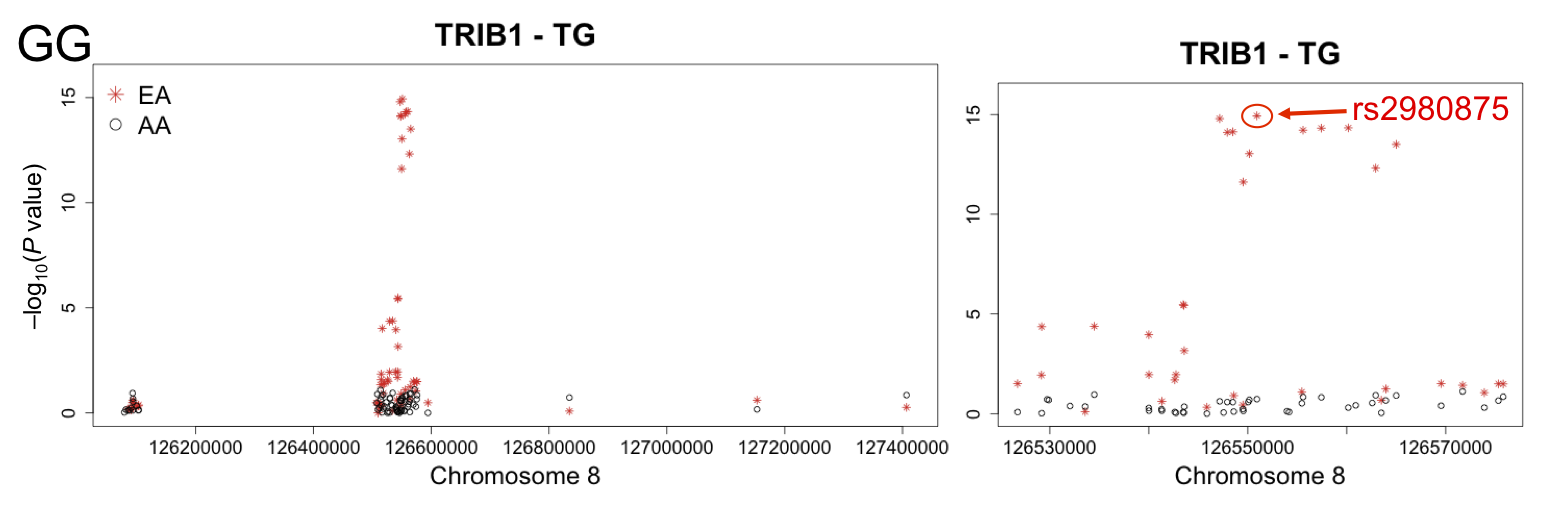

Supplement: Figure S2 — Graphical plots of SNPs in each lipid-associated locus. The x-axis indicates the basepair position on the indicated chromosome. The y-axis indicates the negative log of the P value of association with the indicated lipid trait. Red stars indicate the P values of association of the SNPs in European Americans, black circles in African Americans. Each of the best independently associated SNPs in each locus is circled and labeled. (DOC) [file pone.0036473.s002.doc]
