# Supplementary material for: Multi-Ethnic Analysis of Lipid-Associated Loci: The NHLBI CARe Project
Source: PLoS One. 2012 May 21;7(5):e36473. doi: 10.1371/journal.pone.0036473 (PMC3357427; doi:10.1371/journal.pone.0036473)
Supplement: Table S1 — CARe participant characteristics. (DOC) [file pone.0036473.s003.doc]

**Table S1.** CARe participant characteristics.

| Study | ARIC  AA | CARDIA  AA | CFS  AA | CHS  AA | JHS  AA | MESA  AA | ARIC  EA | CARDIA  EA | CFS  EA | CHS  EA | FHS  EA | MESA  EA |
| --- | --- | --- | --- | --- | --- | --- | --- | --- | --- | --- | --- | --- |
| N (phenotypes) | 4,141 | 2,637 | 721 | 842 | 2,702 | 1,754 | 11,412 | 2,478 | 722 | 4,653 | 10,358 | 2,537 |
| Mean age (y) | 54±6 | 24±4 | 39±20 | 73±6 | 52±13 | 62±10 | 54±6 | 25±3 | 42±20 | 73±6 | 49±15 | 63±10 |
| Female sex (%) | 62 | 56 | 57 | 63 | 62 | 55 | 53 | 53 | 52 | 56 | 54 | 52 |
| Total cholesterol, mg/dl | 215±45 | 177±34 | 171±42 | 210±40 | 197±39 | 190±36 | 215±50 | 176±32 | 181±40 | 211±39 | 204±41 | 196±35 |
| LDL cholesterol, mg/dl | 137±43 | 110±32 | 96±33 | 129±37 | 125±36 | 116±33 | 138±40 | 108±30 | 101±30 | 130±36 | 120±34 | 117±30 |
| HDL cholesterol, mg/dl | 55 ± 18 | 54 ± 13 | 48 ± 15 | 60 ± 16 | 51 ± 15 | 52 ± 15 | 50 ± 17 | 51 ± 13 | 46 ± 13 | 53 ± 16 | 51 ± 15 | 52 ± 16 |
| Triglycerides, mg/dl | 114±81 | 67±38 | 102±62 | 116±63 | 109±98 | 105±69 | 138±93 | 79±57 | 143±118 | 144±79 | 112±95 | 133±90 |
| Lipid lowering therapy (%) | 1 | - | 8 | 7 | 11 | 16 | 3 | - | 8 | 5 | 4 | 18 |

Values with ‘±’ are means ± standard deviation. AA = African American; EA = European American.
