# Supplementary material for: Multi-Ethnic Analysis of Lipid-Associated Loci: The NHLBI CARe Project
Source: PLoS One. 2012 May 21;7(5):e36473. doi: 10.1371/journal.pone.0036473 (PMC3357427; doi:10.1371/journal.pone.0036473)
Supplement: Table S2 — Hardy-Weinberg P values for lipid-associated SNP variants. (DOC) [file pone.0036473.s004.doc]

**Table S2.** Hardy-Weinberg *P* values for lipid-associated SNP variants.

| Gene(s) of interest in associated interval | Trait | SNP | ARIC  EA | CARDIA  EA | CFS  EA | CHS  EA | FHS  EA | MESA  EA | ARIC  AA | CARDIA  AA | CFS  AA | CHS  AA | JHS  AA | MESA  AA |
| --- | --- | --- | --- | --- | --- | --- | --- | --- | --- | --- | --- | --- | --- | --- |
| *PCSK9* | LDL | rs11591147 | 0.7404 | 1 | 0.04464 | 0.2282 | 1 | 1 | 1 | 0.02981 | 1 | 1 | 1 | 1 |
|  |  | rs11806638 | 0.1452 | 0.4935 | 1 | 0.1047 | 0.6223 | 0.7333 | 0.5651 | 0.7488 | 0.8364 | 0.106 | 0.4805 | 0.1021 |
| *ANGPTL3* | TG | rs1748197 | 0.08988 | 0.2515 | 0.2955 | 0.017 | 0.5665 | 0.6972 | 0.1549 | 0.9504 | 0.06009 | 0.7522 | 0.3241 | 0.452 |
| *SORT1* | LDL | rs7528419 | 0.9766 | 0.1975 | 0.1129 | 0.3953 | 0.1624 | 0.3112 | 0.1038 | 0.6728 | 0.195 | 0.7792 | 0.2566 | 0.5684 |
|  |  | rs12740374 | 0.9532 | 0.2242 | 0.1129 | 0.45 | 0.2185 | 0.3414 | 0.08583 | 0.9412 | 0.08492 | 0.3779 | 0.1567 | 0.3213 |
| *GALNT2* | HDL | rs4846918 | 0.253 | 0.6083 | 0.7414 | 0.8502 | 0.9129 | 0.3548 | 0.1855 | 0.3844 | 0.4052 | 0.7643 | 0.4474 | 0.3124 |
| *APOB* | LDL | rs934197 | 0.6573 | 0.8523 | 0.8563 | 0.2173 | 0.8461 | 0.5811 | 0.3936 | 0.8075 | 1 | 0.02734 | 0.1957 | 0.8352 |
|  |  | rs562338 | 0.2867 | 0.9303 | 0.8227 | 0.3425 | 0.3596 | 0.9485 | 0.1483 | 0.3022 | 1 | 1 | 0.4157 | 0.4708 |
|  | HDL | rs673548 | 0.5041 | 0.4836 | 0.7886 | 0.7083 | 0.4322 | 0.751 | 0.3409 | 0.5284 | 0.4855 | 0.5282 | 0.5359 | 0.7115 |
|  | TG | rs1042034 | 0.4847 | 0.5323 | 0.7886 | 0.7437 | 0.4298 | 0.7033 | 0.446 | 0.9176 | 1 | 0.7783 | 1 | 0.8458 |
| *GCKR* | TG | rs1260326 | 0.158 | 0.5841 | 1 | 0.7455 | 0.1324 | 0.703 | 0.6025 | 0.5156 | 0.1892 | 0.5221 | 0.8264 | 0.1242 |
| *ABCG5-ABCG8* | LDL | rs4953023 | 0.4768 | 0.8409 | 1 | 0.061 | 1 | 0.5109 | 0.1605 | 1 | 0.5971 | 0.6178 | 0.8487 | 0.8627 |
| *HMGCR* | LDL | rs12916 | 0.04207 | 0.07409 | 0.7509 | 0.691 | 0.08228 | 0.2435 | 0.3293 | 0.18 | 0.6562 | 0.6436 | 0.2603 | 0.4671 |
| *LPA* | LDL | rs10455872 | 0.04801 | 0.6754 | 0.2802 | 1 | 0.2818 | 0.5246 | 1 | 0.1764 | 0.01149 | 0.1701 | 1 | 1 |
| *NPC1L1* | LDL | rs17725246 | 0.04363 | 0.3036 | 0.6277 | 0.2487 | 0.4031 | 0.2491 | 0.3808 | 0.02565 | 1 | 1 | 0.3764 | 1 |
| *MLXIPL* | TG | rs17145750 | 0.2895 | 0.9213 | 0.2153 | 0.8092 | 0.644 | 0.2091 | 0.8529 | 0.665 | 0.1267 | 0.3553 | 0.002975 | 1 |
| *CD36* | HDL | rs3211938 | 1 | 1 | 1 | 1 | 1 | 1 | 0.7214 | 0.3853 | 0.5813 | 0.6075 | 0.1716 | 0.03108 |
| *LPL* | HDL | rs3916027 | 0.9788 | 0.1364 | 0.8439 | 0.1444 | 0.5668 | 0.7219 | 0.2156 | 0.6389 | 1 | 0.542 | 0.6487 | 0.08594 |
|  |  | rs13702 | 0.7453 | 0.1604 | 0.5804 | 0.09618 | 0.4585 | 0.4457 | 0.1781 | 0.1822 | 0.2936 | 0.9419 | 0.9561 | 0.2133 |
|  | TG | rs3916027 | 0.9788 | 0.1364 | 0.8439 | 0.1444 | 0.5668 | 0.7219 | 0.2156 | 0.6389 | 1 | 0.542 | 0.6487 | 0.08594 |
|  |  | rs327 | 0.9589 | 0.2581 | 0.6976 | 0.1775 | 0.4877 | 0.8039 | 0.2931 | 0.2026 | 0.7063 | 0.8788 | 1 | 0.02198 |
| *TRIB1* | LDL | rs6982636 | 0.6969 | 0.01495 | 1 | 0.8981 | 0.07549 | 0.3132 | 0.3072 | 0.5153 | 1 | 0.2043 | 1 | 1 |
|  | HDL | rs2980880 | 0.4597 | 0.05601 | 1 | 0.9427 | 0.01671 | 0.6711 | 0.3199 | 0.06997 | 0.5705 | 0.1217 | 0.3191 | 0.8656 |
|  | TG | rs2980875 | 0.712 | 0.02627 | 0.7541 | 0.7979 | 0.1371 | 0.3998 | 0.3675 | 0.4784 | 1 | 0.1783 | 0.9037 | 0.8729 |
| *ABCA1* | HDL | rs1883025 | 0.8931 | 0.835 | 1 | 0.513 | 0.03065 | 0.8243 | 0.8716 | 0.8038 | 0.2491 | 0.1084 | 0.662 | 0.4967 |
|  |  | rs2515629 | 0.3132 | 0.7031 | 0.813 | 0.8627 | 0.1883 | 0.6639 | 0.567 | 0.3372 | 0.1971 | 0.5156 | 0.2929 | 0.5883 |
| *FADS1-FADS2-FADS3* | HDL | rs1535 | 0.5052 | 0.3998 | 0.5758 | 0.3825 | 0.4116 | 0.8136 | 0.5724 | 0.7479 | 0.7503 | 0.3741 | 0.9159 | 0.02338 |
| *APOA1-C3-A4-A5* | HDL | rs10750097 | 0.5754 | 0.5878 | 0.6622 | 0.2887 | 0.9339 | 0.3218 | 0.7663 | 0.8195 | 1 | 0.7635 | 0.3366 | 0.682 |
|  | TG | rs2075290 | 0.4336 | 0.005182 | 1 | 0.7255 | 0.5195 | 0.7646 | 0.2141 | 0.004235 | 1 | 1 | 0.5649 | 0.7981 |
|  |  | rs9804646 | 0.9439 | 0.1734 | 1 | 0.9143 | 0.302 | 0.4727 | 0.8126 | 0.3928 | 0.5515 | 0.8684 | 1 | 0.912 |
| *MMAB-MVK* | HDL | rs2075440 | 0.7569 | 0.7117 | 1 | 0.9745 | 0.3914 | 0.102 | 0.1895 | 0.7621 | 0.3004 | 1 | 0.7657 | 0.3406 |
| *LIPC* | HDL | rs2070895 | 0.113 | 0.3115 | 0.2827 | 0.09298 | 0.3973 | 0.05868 | 0.8267 | 0.7383 | 1 | 0.1045 | 0.9123 | 0.5503 |
| *CETP* | HDL | rs17231506 | 0.6912 | 0.7155 | 1 | 0.4473 | 0.5961 | 0.627 | 0.9404 | 0.8357 | 1 | 0.2893 | 0.152 | 0.8457 |
|  |  | rs17231520 | 1 | 1 | 1 | 1 | 1 | 1 | 0.4826 | 0.1459 | 0.3459 | 0.7637 | 0.6725 | 0.1255 |
| *LCAT* | HDL | rs2107369 | 0.03466 | 0.8134 | 0.02077 | 0.07675 | 0.6983 | 0.1123 | 0.4533 | 0.395 | 0.4997 | 0.5667 | 0.9425 | 0.07339 |
|  |  | rs255052 | 0.4909 | 0.6631 | 0.02672 | 0.1378 | 0.7133 | 0.494 | 0.5599 | 0.5037 | 0.0684 | 0.5173 | 0.1198 | 0.9395 |
| *HPR* | LDL | rs2000999 | 0.01982 | 0.8713 | 0.813 | 0.003928 | 0.0001603 | 0.4868 | N/A | N/A | 0.4654 | N/A | N/A | N/A |
| *LIPG* | HDL | rs1943981 | 0.6522 | 0.3176 | 0.5751 | 1 | 0.8205 | 0.5401 | 0.1828 | 0.2122 | 1 | 1 | 0.3502 | 1 |
| *ANGPTL4* | HDL | rs2278236 | 0.2689 | 0.5259 | 0.6396 | 0.7256 | 0.2538 | 0.5023 | 0.01945 | 0.2395 | 0.8612 | 0.2737 | 0.2451 | 0.1334 |
| *ICAM1* | LDL | rs5030359 | 1 | 1 | 1 | 1 | 1 | 1 | 1 | 1 | 1 | 1 | 1 | 1 |
| *LDLR* | LDL | rs6511720 | 0.1091 | 1 | 0.3148 | 0.6031 | 0.5738 | 0.6118 | 0.2179 | 0.9015 | 1 | 0.6692 | 0.4754 | 0.6025 |
| *CSPG3-CILP2-PBX4* | TG | rs3794991 | 0.6626 | 0.2725 | 1 | 0.6702 | 0.1454 | 0.77 | 0.3717 | 0.3598 | 0.3459 | 0.7609 | 0.5125 | 0.1456 |
| *APOE* | LDL | rs12721046 | 0.2939 | 0.1539 | 0.2816 | 0.5295 | 0.8908 | 0.1545 | 0.1128 | 1 | 1 | 1 | 0.6225 | 0.6283 |
|  |  | rs389261 | 1 | 1 | 1 | 1 | 1 | 1 | 0.0009908 | 0.7723 | 0.335 | 0.02281 | 0.03224 | 0.2176 |
|  | HDL | rs12721046 | 0.2939 | 0.1539 | 0.2816 | 0.5295 | 0.8908 | 0.1545 | 0.1128 | 1 | 1 | 1 | 0.6225 | 0.6283 |
|  | TG | rs439401 | 0.4677 | 0.9111 | 0.1816 | 0.5888 | 0.7161 | 0.929 | 0.4179 | 0.8303 | 1 | 0.4859 | 0.9129 | 0.5716 |
|  |  | rs12721054 | 1 | 1 | 1 | 1 | 1 | 1 | 0.8607 | 0.8852 | 0.6045 | 1 | 0.675 | 0.7132 |
| *PLTP* | HDL | rs4810479 | 0.9564 | 0.04563 | 0.3941 | 0.508 | 0.4132 | 0.5974 | 0.3407 | 0.4864 | 0.8543 | 0.8798 | 0.4901 | 0.6389 |
