# Supplementary material for: Multi-Ethnic Analysis of Lipid-Associated Loci: The NHLBI CARe Project
Source: PLoS One. 2012 May 21;7(5):e36473. doi: 10.1371/journal.pone.0036473 (PMC3357427; doi:10.1371/journal.pone.0036473)
Supplement: Table S3 — SNP×SNP interactions between the most significant SNPs at each LDL-C-related locus among European Americans. (DOC) [file pone.0036473.s005.doc]

**Table S3.** SNP × SNP interactions between the most significant SNPs at each LDL-C-related locus among European Americans.

|  | rs10455872 | rs11591147 | rs11806638 | rs12721046 | rs12740374 | rs12916 | rs17725246 | rs2000999 | rs389261 | rs4953023 | rs5030359 | rs562338 | rs6511720 | rs6982636 | rs7528419 | rs934197 |
| --- | --- | --- | --- | --- | --- | --- | --- | --- | --- | --- | --- | --- | --- | --- | --- | --- |
| rs10455872 | X |  |  |  |  |  |  |  |  |  |  |  |  |  |  |  |
| rs11591147 | 0.975 | X |  |  |  |  |  |  |  |  |  |  |  |  |  |  |
| rs11806638 | 0.211 | 0.225 | X |  |  |  |  |  |  |  |  |  |  |  |  |  |
| rs12721046 | 0.857 | 0.104 | 0.933 | X |  |  |  |  |  |  |  |  |  |  |  |  |
| rs12740374 | 0.798 | 0.822 | 0.768 | 0.419 | X |  |  |  |  |  |  |  |  |  |  |  |
| rs12916 | 0.120 | 0.069 | 0.832 | 0.412 | 0.026 | X |  |  |  |  |  |  |  |  |  |  |
| rs17725246 | 0.545 | 0.079 | 0.217 | 0.874 | 0.652 | 0.936 | X |  |  |  |  |  |  |  |  |  |
| rs2000999 | 0.443 | 0.345 | 0.619 | 0.483 | 0.657 | 0.896 | 0.355 | X |  |  |  |  |  |  |  |  |
| rs389261 | X | X | X | X | X | X | X | X | X |  |  |  |  |  |  |  |
| rs4953023 | 0.440 | 0.976 | 0.525 | 0.556 | 0.466 | 0.675 | 0.165 | 0.630 | X | X |  |  |  |  |  |  |
| rs5030359 | X | X | X | X | X | X | X | X | X | X | X |  |  |  |  |  |
| rs562338 | 0.477 | 0.220 | 0.532 | 0.238 | 0.389 | 0.738 | 0.134 | 0.146 | X | 0.968 | X | X |  |  |  |  |
| rs6511720 | 0.930 | 0.117 | 0.794 | 0.490 | 0.375 | 0.074 | 0.806 | 0.889 | X | 0.328 | X | 0.587 | X |  |  |  |
| rs6982636 | 0.512 | 0.230 | 0.580 | 0.922 | 0.896 | 0.523 | 0.934 | 0.686 | X | 0.072 | X | 0.252 | 0.337 | X |  |  |
| rs7528419 | 0.833 | 0.814 | 0.784 | 0.413 | X | 0.026 | 0.676 | 0.649 | X | 0.479 | X | 0.429 | 0.404 | 0.893 | X |  |
| rs934197 | 0.927 | 0.607 | 0.859 | 0.016 | 0.784 | 0.190 | 0.238 | 0.783 | X | 0.312 | X | 0.161 | 0.424 | 0.236 | 0.791 | X |

Values represent *P* values for formal interactions from linear regression analyses that included both SNPs and the interaction test. ■, *P* < 0.05; ■, *P* < 0.01; ■, *P* < 0.005.
