# Supplementary material for: Multi-Ethnic Analysis of Lipid-Associated Loci: The NHLBI CARe Project
Source: PLoS One. 2012 May 21;7(5):e36473. doi: 10.1371/journal.pone.0036473 (PMC3357427; doi:10.1371/journal.pone.0036473)
Supplement: Table S4 — SNP×SNP interactions between the most significant SNPs at each LDL-C-related locus among African Americans. (DOC) [file pone.0036473.s006.doc]

**Table S4.** SNP × SNP interactions between the most significant SNPs at each LDL-C-related locus among African Americans.

| **SNP** | rs4953023 | rs562338 | rs934197 | rs12721046 | rs389261 | rs12740374 | rs7528419 | rs12916 | rs2000999 | rs5030359 | rs6511720 | rs10455872 | rs17725246 | rs11591147 | rs11806638 | rs6982636 |
| --- | --- | --- | --- | --- | --- | --- | --- | --- | --- | --- | --- | --- | --- | --- | --- | --- |
| rs4953023 | X |  |  |  |  |  |  |  |  |  |  |  |  |  |  |  |
| rs562338 | 0.613 | X |  |  |  |  |  |  |  |  |  |  |  |  |  |  |
| rs934197 | 0.062 |  | X |  |  |  |  |  |  |  |  |  |  |  |  |  |
| rs12721046 | 0.377 |  |  | X |  |  |  |  |  |  |  |  |  |  |  |  |
| rs389261 | 0.250 |  |  |  | X |  |  |  |  |  |  |  |  |  |  |  |
| rs12740374 | 0.349 |  |  |  |  | X |  |  |  |  |  |  |  |  |  |  |
| rs7528419 | 0.352 |  |  |  |  |  | X |  |  |  |  |  |  |  |  |  |
| rs12916 | 0.225 |  |  |  |  |  |  | X |  |  |  |  |  |  |  |  |
| rs2000999 | X | X | X | X | X | X | X | X | X |  |  |  |  |  |  |  |
| rs5030359 | 0.593 |  |  |  |  |  |  |  | X | X |  |  |  |  |  |  |
| rs6511720 | 0.681 |  |  |  |  |  |  |  | X |  | X |  |  |  |  |  |
| rs10455872 | 0.569 |  |  |  |  |  |  |  | X |  |  | X |  |  |  |  |
| rs17725246 | 0.197 |  |  |  |  |  |  |  | X |  |  |  | X |  |  |  |
| rs11591147 | 0.806 |  |  |  |  |  |  |  | X |  |  |  |  | X |  |  |
| rs11806638 | 0.918 |  |  |  |  |  |  |  | X |  |  |  |  |  | X |  |
| rs6982636 | 0.656 |  |  |  |  |  |  |  | X |  |  |  |  |  |  | X |

Values represent *P* values for formal interactions from linear regression analyses that included both SNPs and the interaction test. ■, *P* < 0.05; ■, *P* < 0.01; ■, *P* < 0.005.
