# Supplementary material for: Multi-Ethnic Analysis of Lipid-Associated Loci: The NHLBI CARe Project
Source: PLoS One. 2012 May 21;7(5):e36473. doi: 10.1371/journal.pone.0036473 (PMC3357427; doi:10.1371/journal.pone.0036473)
Supplement: Table S5 — SNP×SNP interactions between the most significant SNPs at each HDL-C-related locus among European Americans. (DOC) [file pone.0036473.s007.doc]

**Table S5.** SNP × SNP interactions between the most significant SNPs at each HDL-C-related locus among European Americans.

| **SNP** | rs10750097 | rs12721046 | rs1535 | rs17231506 | rs1883025 | rs1943981 | rs2070895 | rs2075440 | rs2107369 | rs2278236 | rs2980880 | rs3916027 | rs4810479 | rs4846918 | rs673548 |
| --- | --- | --- | --- | --- | --- | --- | --- | --- | --- | --- | --- | --- | --- | --- | --- |
| rs10750097 | X |  |  |  |  |  |  |  |  |  |  |  |  |  |  |
| rs12721046 | 0.174 | X |  |  |  |  |  |  |  |  |  |  |  |  |  |
| rs1535 | 0.766 | 0.516 | X |  |  |  |  |  |  |  |  |  |  |  |  |
| rs17231506 | 0.564 | 0.520 | 0.725 | X |  |  |  |  |  |  |  |  |  |  |  |
| rs1883025 | 0.556 | 0.379 | 0.616 | 0.220 | X |  |  |  |  |  |  |  |  |  |  |
| rs1943981 | 0.625 | 0.199 | 0.262 | 0.900 | 0.490 | X |  |  |  |  |  |  |  |  |  |
| rs2070895 | 0.835 | 0.097 | 0.929 | 0.745 | 0.371 | 0.228 | X |  |  |  |  |  |  |  |  |
| rs2075440 | 0.616 | 0.996 | 0.225 | 0.021 | 0.890 | 0.228 | 0.701 | X |  |  |  |  |  |  |  |
| rs2107369 | 0.193 | 0.937 | 0.744 | 0.110 | 0.560 | 0.109 | 0.154 | 0.474 | X |  |  |  |  |  |  |
| rs2278236 | 0.754 | 0.397 | 0.803 | 0.020 | 0.251 | 0.745 | 0.015 | 0.457 | 0.509 | X |  |  |  |  |  |
| rs2980880 | 0.067 | 0.791 | 0.771 | 0.292 | 0.002 | 0.253 | 0.243 | 0.190 | 0.835 | 0.097 | X |  |  |  |  |
| rs3916027 | 0.941 | 0.022 | 0.022 | 0.104 | 0.053 | 0.980 | 0.426 | 0.238 | 0.892 | 0.178 | 0.845 | X |  |  |  |
| rs4810479 | 0.193 | 0.016 | 0.538 | 0.162 | 0.556 | 0.315 | 0.165 | 0.331 | 0.122 | 0.367 | 0.214 | 0.136 | X |  |  |
| rs4846918 | 0.407 | 0.757 | 0.946 | 0.949 | 0.284 | 0.380 | 0.682 | 0.724 | 0.087 | 0.809 | 0.722 | 0.454 | 0.830 | X |  |
| rs673548 | 0.785 | 0.207 | 0.520 | 0.742 | 0.361 | 0.042 | 0.223 | 0.867 | 0.146 | 0.398 | 0.547 | 0.981 | 0.293 | 0.122 | X |

Values represent *P* values for formal interactions from linear regression analyses that included both SNPs and the interaction test. ■, *P* < 0.05; ■, *P* < 0.01; ■, *P* < 0.005.
