# Supplementary material for: Multi-Ethnic Analysis of Lipid-Associated Loci: The NHLBI CARe Project
Source: PLoS One. 2012 May 21;7(5):e36473. doi: 10.1371/journal.pone.0036473 (PMC3357427; doi:10.1371/journal.pone.0036473)
Supplement: Table S6 — SNP×SNP interactions between the most significant SNPs at each HDL-C-related locus among African Americans. (DOC) [file pone.0036473.s008.doc]

**Table S6.** SNP × SNP interactions between the most significant SNPs at each HDL-C-related locus among African Americans.

| **SNP** | rs2515629 | rs3211938 | rs17231520 | rs255052 | rs2070895 | rs13702 |
| --- | --- | --- | --- | --- | --- | --- |
| rs2515629 | X |  |  |  |  |  |
| rs3211938 | 0.345 | X |  |  |  |  |
| rs17231520 | 0.945 |  | X |  |  |  |
| rs255052 | 0.385 |  |  | X |  |  |
| rs2070895 | 0.293 |  |  |  | X |  |
| rs13702 | 0.048 |  |  |  |  | X |

Values represent *P* values for formal interactions from linear regression analyses that included both SNPs and the interaction test. ■, *P* < 0.05; ■, *P* < 0.01; ■, *P* < 0.005.
