# Supplementary material for: Multi-Ethnic Analysis of Lipid-Associated Loci: The NHLBI CARe Project
Source: PLoS One. 2012 May 21;7(5):e36473. doi: 10.1371/journal.pone.0036473 (PMC3357427; doi:10.1371/journal.pone.0036473)
Supplement: Table S7 — SNP×SNP interactions between the most significant SNPs at each triglyceride-related locus among European Americans. (DOC) [file pone.0036473.s009.doc]

**Table S7.** SNP × SNP interactions between the most significant SNPs at each triglyceride-related locus among European Americans.

| **SNP** | rs1042034 | rs1260326 | rs12721054 | rs17145750 | rs1748197 | rs2075290 | rs2980875 | rs327 | rs3794991 | rs3916027 | rs439401 | rs9804646 |
| --- | --- | --- | --- | --- | --- | --- | --- | --- | --- | --- | --- | --- |
| rs1042034 | X |  |  |  |  |  |  |  |  |  |  |  |
| rs1260326 | 0.024 | X |  |  |  |  |  |  |  |  |  |  |
| rs12721054 | 0.200 | 0.958 | X |  |  |  |  |  |  |  |  |  |
| rs17145750 | 0.630 | 0.987 | 0.576 | X |  |  |  |  |  |  |  |  |
| rs1748197 | 0.281 | 0.935 | 0.090 | 0.009 | X |  |  |  |  |  |  |  |
| rs2075290 | 0.421 | 0.955 | 0.789 | 0.121 | 0.181 | X |  |  |  |  |  |  |
| rs2980875 | 0.702 | 0.690 | 0.397 | 0.263 | 0.310 | 0.295 | X |  |  |  |  |  |
| rs327 | 0.283 | 0.089 | 0.033 | 0.641 | 0.505 | 0.179 | 0.623 | X |  |  |  |  |
| rs3794991 | 0.293 | 0.415 | 0.045 | 0.927 | 0.041 | 0.417 | 0.323 | 0.691 | X |  |  |  |
| rs3916027 | 0.327 | 0.117 | 0.032 | 0.514 | 0.386 | 0.158 | 0.790 | 0.894 | 0.965 | X |  |  |
| rs439401 | 0.941 | 0.854 | 0.281 | 0.113 | 0.890 | 0.412 | 0.927 | 0.990 | 0.956 | 0.900 | X |  |
| rs9804646 | 0.414 | 0.030 | 0.581 | 0.332 | 0.439 | 0.214 | 0.026 | 0.730 | 0.687 | 0.587 | 0.600 | X |

Values represent *P* values for formal interactions from linear regression analyses that included both SNPs and the interaction test. ■, *P* < 0.05; ■, *P* < 0.01; ■, *P* < 0.005.
