# Supplementary material for: Multi-Ethnic Analysis of Lipid-Associated Loci: The NHLBI CARe Project
Source: PLoS One. 2012 May 21;7(5):e36473. doi: 10.1371/journal.pone.0036473 (PMC3357427; doi:10.1371/journal.pone.0036473)
Supplement: Table S8 — SNP×SNP interactions between the most significant SNPs at each triglyceride-related locus among African Americans. (DOC) [file pone.0036473.s010.doc]

**Table S8.** SNP × SNP interactions between the most significant SNPs at each triglyceride-related locus among African Americans.

| **SNP** | rs1042034 | rs1260326 | rs12721054 | rs17145750 | rs1748197 | rs2075290 | rs2980875 | rs327 | rs3794991 | rs3916027 | rs439401 | rs9804646 |
| --- | --- | --- | --- | --- | --- | --- | --- | --- | --- | --- | --- | --- |
| rs1042034 | X |  |  |  |  |  |  |  |  |  |  |  |
| rs1260326 | 0.035 | X |  |  |  |  |  |  |  |  |  |  |
| rs12721054 | 0.521 | 0.477 | X |  |  |  |  |  |  |  |  |  |
| rs17145750 | 0.994 | 0.600 | 0.493 | X |  |  |  |  |  |  |  |  |
| rs1748197 | 0.227 | 0.936 | 0.192 | 0.777 | X |  |  |  |  |  |  |  |
| rs2075290 | 0.494 | 0.203 | 0.440 | 0.589 | 0.012 | X |  |  |  |  |  |  |
| rs2980875 | 0.438 | 0.405 | 0.088 | 0.526 | 0.296 | 0.825 | X |  |  |  |  |  |
| rs327 | 0.023 | 0.891 | 0.550 | 0.270 | 0.904 | 0.152 | 0.456 | X |  |  |  |  |
| rs3794991 | 0.003 | 0.270 | 0.450 | 0.862 | 0.158 | 0.085 | 0.421 | 0.874 | X |  |  |  |
| rs3916027 | 0.046 | 0.899 | 0.699 | 0.508 | 0.870 | 0.047 | 0.229 | 0.946 | 0.812 | X |  |  |
| rs439401 | 0.969 | 0.366 | 0.869 | 0.396 | 0.842 | 0.697 | 0.158 | 0.376 | 0.872 | 0.565 | X |  |
| rs9804646 | 0.998 | 0.282 | 0.087 | 0.094 | 0.334 | 0.348 | 0.039 | 0.123 | 0.249 | 0.502 | 0.710 | X |

Values represent *P* values for formal interactions from linear regression analyses that included both SNPs and the interaction test. ■, *P* < 0.05; ■, *P* < 0.01; ■, *P* < 0.005.
