# Supplementary material for: Multi-Ethnic Analysis of Lipid-Associated Loci: The NHLBI CARe Project
Source: PLoS One. 2012 May 21;7(5):e36473. doi: 10.1371/journal.pone.0036473 (PMC3357427; doi:10.1371/journal.pone.0036473)
Supplement: Table S9 — SNP×sex interaction tests for the most significant SNPs at each LDL-C-related locus. (DOC) [file pone.0036473.s011.doc]

**Table S9.** SNP × sex interaction tests for the most significant SNPs at each LDL-C-related locus.

|  |  | **European Americans** | | | | | |  | **African Americans** | | | | |
| --- | --- | --- | --- | --- | --- | --- | --- | --- | --- | --- | --- | --- | --- |
| **Locus** | **SNP** |  | **Men** |  | **Women** |  | **Interaction** |  | **Men** |  | **Women** |  | **Interaction** |
|  |  |  | ***P*** |  | ***P*** |  | ***P*** |  | ***P*** |  | ***P*** |  | ***P*** |
| *ABCG5-ABCG8* | rs4953023 |  | 8.622E-04 |  | 6.416E-06 |  | 4.702E-01 |  | 2.394E-02 |  | 2.218E-02 |  | 7.942E-01 |
| *APOB* | rs562338 |  | 3.805E-16 |  | 9.281E-21 |  | 6.164E-01 |  | 5.094E-06 |  | 3.465E-04 |  | 1.409E-01 |
| *APOB* | rs934197 |  | 3.114E-14 |  | 8.507E-24 |  | 2.138E-01 |  | 9.360E-02 |  | 4.890E-02 |  | 8.218E-01 |
| *APOE* | rs12721046 |  | 1.313E-12 |  | 2.027E-19 |  | 3.155E-01 |  | 2.225E-02 |  | 8.905E-03 |  | 7.671E-01 |
| *APOE* | rs389261 |  | — |  | — |  | — |  | 3.226E-07 |  | 2.924E-09 |  | 8.636E-01 |
| *SORT1* | rs12740374 |  | 7.467E-25 |  | 5.059E-31 |  | 6.352E-01 |  | 9.640E-09 |  | 3.344E-13 |  | 9.163E-01 |
| *SORT1* | rs7528419 |  | 6.278E-25 |  | 3.437E-31 |  | 6.313E-01 |  | 1.607E-06 |  | 4.135E-12 |  | 5.109E-01 |
| *HMGCR* | rs12916 |  | 1.336E-07 |  | 2.225E-07 |  | 8.110E-01 |  | 2.531E-01 |  | 2.177E-01 |  | 6.515E-01 |
| *HPR* | rs2000999 |  | 1.633E-06 |  | 4.911E-04 |  | 2.425E-01 |  | — |  | — |  | — |
| *ICAM1* | rs5030359 |  | — |  | — |  | — |  | 5.907E-05 |  | 3.022E-05 |  | 5.601E-01 |
| *LDLR* | rs6511720 |  | 3.067E-25 |  | 7.510E-29 |  | 8.381E-01 |  | 2.021E-06 |  | 1.505E-14 |  | 2.178E-01 |
| *LPA* | rs10455872 |  | 7.614E-05 |  | 1.953E-09 |  | 2.143E-01 |  | 8.192E-01 |  | 1.484E-01 |  | 3.356E-01 |
| *NPC1L1* | rs17725246 |  | 8.223E-06 |  | 2.450E-03 |  | 2.040E-01 |  | 2.135E-03 |  | 6.300E-01 |  | 2.451E-02 |
| *PCSK9* | rs11591147 |  | 8.153E-18 |  | 2.744E-14 |  | 2.373E-01 |  | 8.053E-03 |  | 5.894E-03 |  | 4.348E-01 |
| *PCSK9* | rs11806638 |  | 8.620E-02 |  | 7.979E-02 |  | 8.829E-01 |  | 3.079E-05 |  | 1.083E-07 |  | 8.853E-01 |
| *TRIB1* | rs6982636 |  | 8.321E-05 |  | 6.373E-06 |  | 9.544E-01 |  | 8.459E-01 |  | 8.987E-01 |  | 7.442E-01 |

*P* values for men and women generated from linear regression models for each SNP that included only male or female participants, respectively. Interaction *P* values generated from a formal interaction test of SNP × sex, included as part of logistic regression models that included all participants and contained SNP, sex, and SNP × sex as predictor variables of LDL-C.
