# Supplementary material for: Multi-Ethnic Analysis of Lipid-Associated Loci: The NHLBI CARe Project
Source: PLoS One. 2012 May 21;7(5):e36473. doi: 10.1371/journal.pone.0036473 (PMC3357427; doi:10.1371/journal.pone.0036473)
Supplement: Table S10 — SNP×sex interaction tests for the most significant SNPs at each HDL-C-related locus. (DOC) [file pone.0036473.s012.doc]

**Table S10.** SNP × sex interaction tests for the most significant SNPs at each HDL-C-related locus.

|  |  | **European Americans** | | | | | |  | **African Americans** | | | | |
| --- | --- | --- | --- | --- | --- | --- | --- | --- | --- | --- | --- | --- | --- |
| **Locus** | **SNP** |  | **Males** |  | **Females** |  | **Interaction** |  | **Males** |  | **Females** |  | **Interaction** |
|  |  |  | ***P*** |  | ***P*** |  | ***P*** |  | ***P*** |  | ***P*** |  | ***P*** |
| *ABCA1* | rs1883025 |  | 4.454E-05 |  | 1.457E-03 |  | 5.376E-01 |  | 4.579E-01 |  | 1.903E-02 |  | 3.186E-01 |
| *ABCA1* | rs2515629 |  | 4.679E-01 |  | 1.154E-01 |  | 7.549E-02 |  | 1.355E-03 |  | 2.518E-05 |  | 7.397E-01 |
| *ANGPTL4* | rs2278236 |  | 1.901E-03 |  | 5.802E-05 |  | 5.938E-01 |  | 4.775E-01 |  | 6.119E-01 |  | 4.324E-01 |
| *APOA1-C3-A4-A5* | rs10750097 |  | 5.130E-12 |  | 3.167E-09 |  | 4.483E-01 |  | 1.522E-01 |  | 7.489E-01 |  | 1.963E-01 |
| *APOB* | rs673548 |  | 7.993E-05 |  | 1.105E-05 |  | 9.802E-01 |  | 3.454E-01 |  | 6.988E-02 |  | 6.831E-01 |
| *APOE* | rs12721046 |  | 5.644E-04 |  | 3.367E-05 |  | 7.739E-01 |  | 2.613E-01 |  | 7.638E-02 |  | 8.710E-01 |
| *CD36* | rs3211938 |  | 3.580E-02 |  | 2.968E-01 |  | 2.503E-01 |  | 1.401E-04 |  | 1.366E-09 |  | 5.378E-01 |
| *CETP* | rs17231506 |  | 1.131E-60 |  | 6.831E-68 |  | 8.422E-01 |  | 4.229E-06 |  | 1.500E-04 |  | 7.018E-01 |
| *CETP* | rs17231520 |  | - |  | - |  | - |  | 6.540E-32 |  | 4.206E-31 |  | 3.710E-01 |
| *FADS1-FADS2-FADS3* | rs1535 |  | 6.800E-04 |  | 7.600E-07 |  | 2.863E-01 |  | 2.484E-02 |  | 3.266E-03 |  | 8.712E-01 |
| *GALNT2* | rs4846918 |  | 3.492E-03 |  | 1.903E-05 |  | 4.330E-01 |  | 3.822E-01 |  | 6.300E-01 |  | 8.911E-01 |
| *LCAT* | rs2107369 |  | 1.339E-03 |  | 9.255E-06 |  | 3.620E-01 |  | 9.785E-01 |  | 5.025E-01 |  | 9.749E-01 |
| *LCAT* | rs255052 |  | 5.804E-03 |  | 1.286E-05 |  | 2.246E-01 |  | 5.320E-05 |  | 1.536E-05 |  | 6.014E-01 |
| *LIPC* | rs2070895 |  | 8.283E-12 |  | 3.565E-15 |  | 6.514E-01 |  | 6.291E-07 |  | 1.538E-05 |  | 2.352E-01 |
| *LIPG* | rs1943981 |  | 2.290E-06 |  | 1.808E-09 |  | 4.759E-01 |  | 6.264E-01 |  | 5.033E-01 |  | 7.936E-01 |
| *LPL* | rs13702 |  | 5.606E-16 |  | 4.724E-16 |  | 8.222E-01 |  | 2.536E-04 |  | 1.966E-05 |  | 6.035E-01 |
| *LPL* | rs3916027 |  | 1.426E-17 |  | 4.367E-18 |  | 8.936E-01 |  | 5.438E-03 |  | 6.229E-06 |  | 6.281E-01 |
| *MMAB-MVK* | rs2075440 |  | 5.117E-05 |  | 8.894E-04 |  | 5.520E-01 |  | 6.169E-01 |  | 7.389E-01 |  | 6.742E-01 |
| *PLTP* | rs4810479 |  | 1.230E-01 |  | 7.114E-12 |  | 1.512E-04 |  | 1.329E-03 |  | 1.202E-02 |  | 6.132E-01 |
| *TRIB1* | rs2980880 |  | 2.333E-04 |  | 3.791E-04 |  | 7.776E-01 |  | 9.252E-01 |  | 3.741E-01 |  | 6.416E-01 |

*P* values for men and women generated from linear regression models for each SNP that included only male or female participants, respectively. Interaction *P* values generated from a formal interaction test of SNP × sex, included as part of logistic regression models that included all participants and contained SNP, sex, and SNP × sex as predictor variables of HDL-C.
