# Supplementary material for: Multi-Ethnic Analysis of Lipid-Associated Loci: The NHLBI CARe Project
Source: PLoS One. 2012 May 21;7(5):e36473. doi: 10.1371/journal.pone.0036473 (PMC3357427; doi:10.1371/journal.pone.0036473)
Supplement: Table S11 — SNP×sex interaction tests for the most significant SNPs at each triglyceride-related locus. (DOC) [file pone.0036473.s013.doc]

**Table S11.** SNP × sex interaction tests for the most significant SNPs at each triglyceride-related locus.

|  |  | **European Americans** | | | | | |  | **African Americans** | | | | |
| --- | --- | --- | --- | --- | --- | --- | --- | --- | --- | --- | --- | --- | --- |
| **Locus** | **SNP** |  | **Males** |  | **Females** |  | **Interaction** |  | **Males** |  | **Females** |  | **Interaction** |
|  |  |  | ***P*** |  | ***P*** |  | ***P*** |  | ***P*** |  | ***P*** |  | ***P*** |
| *ANGPTL3* | rs1748197 |  | 8.205E-05 |  | 5.509E-04 |  | 6.059E-01 |  | 2.974E-01 |  | 9.419E-03 |  | 4.875E-01 |
| *APOA1-C3-A4-A5* | rs2075290 |  | 1.748E-33 |  | 6.071E-25 |  | 1.117E-01 |  | 4.216E-02 |  | 5.167E-03 |  | 7.951E-01 |
| *APOA1-C3-A4-A5* | rs9804646 |  | 7.530E-04 |  | 5.825E-01 |  | 2.182E-02 |  | 2.210E-04 |  | 2.307E-04 |  | 5.843E-01 |
| *APOB* | rs1042034 |  | 1.539E-05 |  | 9.707E-07 |  | 9.360E-01 |  | 7.301E-02 |  | 1.756E-03 |  | 6.323E-01 |
| *APOE* | rs12721054 |  | 7.506E-01 |  | 5.526E-01 |  | 7.996E-01 |  | 1.147E-14 |  | 3.167E-14 |  | 2.764E-01 |
| *APOE* | rs439401 |  | 3.781E-15 |  | 8.112E-05 |  | 1.289E-03 |  | 5.648E-01 |  | 7.948E-01 |  | 5.263E-01 |
| *CSPG3-CILP2-PBX4* | rs3794991 |  | 4.410E-06 |  | 5.686E-06 |  | 6.936E-01 |  | 7.607E-01 |  | 8.147E-01 |  | 6.890E-01 |
| *GCKR* | rs1260326 |  | 1.274E-18 |  | 1.749E-20 |  | 8.236E-01 |  | 4.811E-02 |  | 2.168E-04 |  | 4.392E-01 |
| *LPL* | rs327 |  | 1.005E-21 |  | 7.336E-19 |  | 4.576E-01 |  | 3.255E-05 |  | 2.964E-06 |  | 7.222E-01 |
| *LPL* | rs3916027 |  | 1.474E-23 |  | 9.432E-22 |  | 5.541E-01 |  | 4.894E-05 |  | 5.028E-05 |  | 4.957E-01 |
| *MLXIPL* | rs17145750 |  | 1.123E-15 |  | 5.657E-13 |  | 2.974E-01 |  | 6.401E-01 |  | 1.840E-02 |  | 4.688E-02 |
| *TRIB1* | rs2980875 |  | 9.607E-11 |  | 1.050E-07 |  | 2.276E-01 |  | 2.551E-01 |  | 4.415E-01 |  | 6.778E-01 |

*P* values for men and women generated from linear regression models for each SNP that included only male or female participants, respectively. Interaction *P* values generated from a formal interaction test of SNP × sex, included as part of logistic regression models that included all participants and contained SNP, sex, and SNP × sex as predictor variables of triglycerides.
