# Supplementary material for: Multi-Ethnic Analysis of Lipid-Associated Loci: The NHLBI CARe Project
Source: PLoS One. 2012 May 21;7(5):e36473. doi: 10.1371/journal.pone.0036473 (PMC3357427; doi:10.1371/journal.pone.0036473)
Supplement: Table S12 — Top ten significant SNP×sex interactions for each lipid trait among European Americans. (DOC) [file pone.0036473.s014.doc]

**Table S12.** Top ten significant SNP × sex interactions for each lipid trait among European Americans.

|  |  | **Alleles** | |  |  |  | **Individual GC Only** | |  | **Overall GC Correction** | |
| --- | --- | --- | --- | --- | --- | --- | --- | --- | --- | --- | --- |
|  | **SNP** | **1** | **2** | **A1 Freq.** | ***N*** |  | **Direction** | ***P*** |  | **Direction** | ***P*** |
|  |  |  |  |  |  |  |  |  |  |  |  |
| **LDL-C** | |  |  |  |  |  |  |  |  |  |  |
|  |  |  |  |  |  |  |  |  |  |  |  |
| 1 | rs3741379 | T | G | 0.0003 | 10846 |  | -+???? | 7.46E-10 |  | - | 8.85E-10 |
| 2 | rs3219101 | A | G | 0.9984 | 6318 |  | ????-? | 1.33E-07 |  | - | 1.51E-07 |
| 3 | rs6679302 | A | G | 0.001 | 23316 |  | ++?++- | 3.28E-06 |  | + | 3.63E-06 |
| 4 | rs6000 | A | C | 0.9997 | 19609 |  | -??--? | 4.71E-06 |  | - | 5.19E-06 |
| 5 | rs5999 | T | C | 0.9997 | 19619 |  | -??--? | 1.54E-05 |  | - | 1.68E-05 |
| 6 | rs17549671 | A | G | 0.9997 | 19610 |  | -??--? | 1.55E-05 |  | - | 1.69E-05 |
| 7 | rs11784828 | T | G | 0.0002 | 9413 |  | +????? | 1.63E-05 |  | + | 1.77E-05 |
| 8 | rs12120605 | T | G | 0.0864 | 23582 |  | ++++++ | 1.77E-05 |  | + | 1.93E-05 |
| 9 | rs5030344 | A | G | 0.0003 | 3867 |  | ???-?? | 2.72E-05 |  | - | 2.95E-05 |
| 10 | rs9332618 | A | G | 0.1117 | 23584 |  | +++++- | 3.03E-05 |  | + | 3.28E-05 |
|  |  |  |  |  |  |  |  |  |  |  |  |
| **HDL-C** | |  |  |  |  |  |  |  |  |  |  |
|  |  |  |  |  |  |  |  |  |  |  |  |
| 1 | rs2243309 | C | G | 0.9992 | 6956 |  | ????-? | 2.19E-08 |  | - | 2.19E-08 |
| 2 | rs11658081 | T | G | 0.0004 | 13508 |  | +??+?? | 6.67E-06 |  | + | 6.67E-06 |
| 3 | rs6065904 | A | G | 0.2165 | 24790 |  | ++-+++ | 7.53E-06 |  | + | 7.53E-06 |
| 4 | rs2242501 | A | G | 0.2612 | 24789 |  | ++--++ | 2.42E-05 |  | + | 2.42E-05 |
| 5 | rs6073952 | A | G | 0.1904 | 24778 |  | ++-+++ | 3.04E-05 |  | + | 3.04E-05 |
| 6 | rs35404985 | A | G | 0.0002 | 16533 |  | +???-? | 4.08E-05 |  | + | 4.08E-05 |
| 7 | rs771722 | A | G | 0.7409 | 24774 |  | ++++++ | 4.43E-05 |  | + | 4.43E-05 |
| 8 | rs137206 | T | C | 0.617 | 24791 |  | ------ | 1.11E-04 |  | - | 1.11E-04 |
| 9 | rs4758287 | A | C | 0.2894 | 24783 |  | ++--++ | 1.29E-04 |  | + | 1.29E-04 |
| 10 | rs17277288 | T | C | 0.9886 | 23165 |  | +?++++ | 1.29E-04 |  | + | 1.29E-04 |
| 11 | rs4810479 | T | C | 0.7442 | 24667 |  | ------ | 1.51E-04 |  | - | 1.51E-04 |
|  |  |  |  |  |  |  |  |  |  |  |  |
| **Triglycerides** | |  |  |  |  |  |  |  |  |  |  |
|  |  |  |  |  |  |  |  |  |  |  |  |
| 1 | rs1248052 | A | C | 0.4508 | 23895 |  | ++-+++ | 4.38E-05 |  | + | 4.38E-05 |
| 2 | rs28944185 | A | G | 0.0002 | 15948 |  | +???+? | 8.81E-05 |  | + | 8.81E-05 |
| 3 | rs10270964 | T | C | 0.9395 | 23882 |  | -+---- | 8.97E-05 |  | - | 8.97E-05 |
| 4 | rs919041 | T | C | 0.927 | 23870 |  | -++--- | 9.01E-05 |  | - | 9.01E-05 |
| 5 | rs17128307 | T | C | 0.1181 | 23898 |  | --+--- | 9.09E-05 |  | - | 9.09E-05 |
| 6 | rs2442719 | T | C | 0.5643 | 23772 |  | ------ | 9.28E-05 |  | - | 9.28E-05 |
| 7 | rs17161334 | T | C | 0.0395 | 23899 |  | -----+ | 1.58E-04 |  | - | 1.58E-04 |
| 8 | rs6063652 | T | C | 0.2608 | 23898 |  | ------ | 1.87E-04 |  | - | 1.87E-04 |
| 9 | rs11674483 | T | C | 0.3401 | 23898 |  | ++-+++ | 2.79E-04 |  | + | 2.79E-04 |
| 10 | rs7309941 | T | C | 0.1476 | 23884 |  | ------ | 3.00E-04 |  | - | 3.00E-04 |
|  |  |  |  |  |  |  |  |  |  |  |  |
