# Supplementary material for: Multi-Ethnic Analysis of Lipid-Associated Loci: The NHLBI CARe Project
Source: PLoS One. 2012 May 21;7(5):e36473. doi: 10.1371/journal.pone.0036473 (PMC3357427; doi:10.1371/journal.pone.0036473)
Supplement: Table S13 — Top ten significant SNP×sex interactions for each lipid trait among African Americans. (DOC) [file pone.0036473.s015.doc]

**Table S13.** Top ten significant SNP × sex interactions for each lipid trait among African Americans.

|  |  | **Alleles** | |  |  |  | **Individual GC Only** | |  | **Overall GC Correction** | |
| --- | --- | --- | --- | --- | --- | --- | --- | --- | --- | --- | --- |
|  | **SNP** | **1** | **2** | **A1 Freq.** | ***N*** |  | **Direction** | ***P*** |  | **Direction** | ***P*** |
|  |  |  |  |  |  |  |  |  |  |  |  |
| **LDL-C** | |  |  |  |  |  |  |  |  |  |  |
|  |  |  |  |  |  |  |  |  |  |  |  |
| 1 | rs9814648 | A | G | 0.3746 | 8853 |  | --+--- | 4.91E-06 |  | - | 4.91E-06 |
| 2 | rs17676277 | A | T | 0.0777 | 8851 |  | ++++++ | 9.60E-06 |  | + | 9.60E-06 |
| 3 | rs6891658 | A | G | 0.7681 | 8857 |  | ---+-- | 1.32E-05 |  | - | 1.32E-05 |
| 4 | rs6576431 | A | G | 0.5622 | 8857 |  | ------ | 1.86E-05 |  | - | 1.86E-05 |
| 5 | rs7664413 | T | C | 0.3271 | 8840 |  | ------ | 3.81E-05 |  | - | 3.81E-05 |
| 6 | rs7091020 | A | G | 0.1394 | 8857 |  | ++++++ | 4.97E-05 |  | + | 4.97E-05 |
| 7 | rs2112650 | A | C | 0.2945 | 8857 |  | --+--- | 0.000106 |  | - | 1.06E-04 |
| 8 | rs215987 | T | C | 0.4929 | 8852 |  | ------ | 0.000158 |  | - | 1.58E-04 |
| 9 | rs7677445 | A | G | 0.8453 | 5848 |  | ?+++++ | 0.000182 |  | + | 1.82E-04 |
| 10 | rs11156577 | T | C | 0.5205 | 8853 |  | ++++++ | 0.000192 |  | + | 1.92E-04 |
|  |  |  |  |  |  |  |  |  |  |  |  |
| **HDL-C** | |  |  |  |  |  |  |  |  |  |  |
|  |  |  |  |  |  |  |  |  |  |  |  |
| 1 | rs12709888 | A | G | 0.0003 | 2891 |  | +????? | 5.81E-07 |  | + | 1.18E-06 |
| 2 | rs974828 | T | C | 0.7735 | 9164 |  | ++++++ | 1.64E-06 |  | + | 3.14E-06 |
| 3 | rs3917030 | A | T | 0.9972 | 9160 |  | ------ | 6.81E-06 |  | - | 1.21E-05 |
| 4 | rs351359 | A | G | 0.4106 | 9168 |  | ++++++ | 3.57E-05 |  | + | 5.84E-05 |
| 5 | rs3212209 | A | G | 0.0115 | 9089 |  | --++-- | 6.65E-05 |  | - | 1.05E-04 |
| 6 | rs11187515 | T | C | 0.1444 | 9177 |  | ------ | 7.87E-05 |  | - | 1.23E-04 |
| 7 | rs10925401 | T | C | 0.6819 | 9176 |  | ++-+++ | 7.99E-05 |  | + | 1.25E-04 |
| 8 | rs8033152 | T | C | 0.2336 | 9178 |  | ++++++ | 8.22E-05 |  | + | 1.29E-04 |
| 9 | rs32574 | A | C | 0.4931 | 7374 |  | ++-++? | 8.71E-05 |  | + | 1.36E-04 |
| 10 | rs4479171 | T | C | 0.8367 | 9128 |  | -+---- | 0.000177 |  | - | 2.67E-04 |
|  |  |  |  |  |  |  |  |  |  |  |  |
| **Triglycerides** | |  |  |  |  |  |  |  |  |  |  |
|  |  |  |  |  |  |  |  |  |  |  |  |
| 1 | rs16148 | T | C | 0.4659 | 8922 |  | ++++++ | 2.59E-06 |  | + | 2.59E-06 |
| 2 | rs12924788 | T | C | 0.7153 | 8914 |  | ------ | 6.21E-06 |  | - | 6.21E-06 |
| 3 | rs9332570 | C | G | 0.3099 | 8922 |  | ++++++ | 1.28E-05 |  | + | 1.28E-05 |
| 4 | rs1894701 | T | C | 0.3098 | 8922 |  | ++++++ | 1.33E-05 |  | + | 1.33E-05 |
| 5 | rs7312807 | T | C | 0.8108 | 8863 |  | ------ | 1.76E-05 |  | - | 1.76E-05 |
| 6 | rs3891250 | T | G | 0.3016 | 8923 |  | --+--- | 1.85E-05 |  | - | 1.85E-05 |
| 7 | rs7226991 | A | G | 0.3692 | 8923 |  | ------ | 2.39E-05 |  | - | 2.39E-05 |
| 8 | rs9974796 | C | G | 0.7491 | 8923 |  | --+--- | 3.17E-05 |  | - | 3.17E-05 |
| 9 | rs6678795 | A | G | 0.7631 | 8922 |  | ------ | 3.84E-05 |  | - | 3.84E-05 |
| 10 | rs7548857 | T | C | 0.7634 | 8923 |  | ------ | 4.17E-05 |  | - | 4.17E-05 |
|  |  |  |  |  |  |  |  |  |  |  |  |
